# The Frequency but not the Phenotype of Circulating Peripheral T Helper Cells is Increased at Later Stages of Progression to Type 1 Diabetes

**DOI:** 10.1002/eji.202451704

**Published:** 2025-06-20

**Authors:** Anna‐Mari Schroderus, Andrea Hanel, Céline Vandamme, Viola Pitkänen, Marja Rytkönen‐Nissinen, Merja Heinäniemi, Mikael Knip, Riitta Veijola, Jorma Toppari, Jorma Ilonen, Johanna Lempainen, Tuure Kinnunen

**Affiliations:** ^1^ Department of Clinical Microbiology Institute of Clinical Medicine University of Eastern Finland Kuopio Finland; ^2^ Faculty of Health Sciences Institute of Biomedicine University of Eastern Finland Kuopio Finland; ^3^ Tampere Center for Child Health Research Tampere University Hospital Tampere Finland; ^4^ Research Program for Clinical and Molecular Metabolism Faculty of Medicine University of Helsinki Helsinki Finland; ^5^ Research Unit of Clinical Medicine Department of Pediatrics Medical Research Center Oulu University Hospital and University of Oulu Oulu Finland; ^6^ Department of Pediatrics University of Turku and Turku University Hospital Turku Finland; ^7^ Research Centre for Integrative Physiology and Pharmacology Centre for Population Health Research InFLAMES Research Flagship Institute of Biomedicine University of Turku Turku Finland; ^8^ Immunogenetics Laboratory Institute of Biomedicine University of Turku Turku Finland; ^9^ ISLAB Laboratory Centre Kuopio Finland

**Keywords:** autoimmunity, B cell‐helper T cells, T cells, T follicular helper, T peripheral helper, Type 1 diabetes

## Abstract

Circulating follicular (cTfh) and peripheral (cTph) T helper cells have been demonstrated to be expanded in several autoimmune diseases, including type 1 diabetes (T1D). Here, we examined the frequencies and phenotypes of these cells at different stages of T1D development and addressed their phenotypic and clonal relationships by analyzing samples from 27 children with newly diagnosed T1D, 29 autoantibody‐positive (AAb^+^) children who later progressed to T1D and 57 healthy, age‐matched controls. Higher frequencies of cTph cells were detected in children with T1D and AAb^+^ children by flow cytometry, but no phenotypic alterations compared with cTph cells from healthy children were observed. Through a single‐cell multiomics approach, we demonstrate that cTph cells appear phenotypically more heterogeneous compared with cTfh cells and that they exhibit phenotypic and clonal sharing with both cTfh as well as CXCR5^−^PD‐1^lo^ memory T cells. Finally, the frequencies of cTph or cTfh cells did not differ in 17 children analyzed during seroconversion for T1D‐associated autoantibodies, the earliest detectable time point for autoimmunity. Collectively, our data demonstrate that cTph cells are a highly heterogeneous population partially sharing features with cTfh cells and that their frequency but not phenotype is altered at later stages of progression to clinical T1D.

## Graphical abstract

Frequencies of circulating T peripheral helper (cTph) and activated T follicular helper (cTfh) cells increase close to the diagnosis of clinical type 1 diabetes, but not at the onset of autoimmunity. Single‐cell analysis reveals cTph as a heterogeneous cell population, sharing features both with cTfh and other memory T cells.

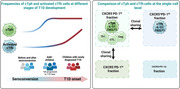

**Abbreviations**
AAb+: autoantibody‐positive
cTfh: circulating follicular T helper
cTph: circulating peripheral T helper
RA: rheumatoid arthritis
SLE: systemic lupus erythematosus
T1D: type 1 diabetes

## 1 Introduction

Type 1 diabetes (T1D) is an autoimmune disease that leads to progressive immune‐mediated destruction of insulin‐producing β‐cells in the pancreas [1]. The clinical presentation of T1D is preceded by a phase during which T1D‐associated islet autoantibodies can be detected in the circulation. Consequently, children positive for multiple persisting autoantibodies (AAb^+^) are at a high risk for developing T1D [2]. The production of antibodies, including autoantibodies, is highly dependent on the help provided by specialized T cells. Of these T cells, the most critical are follicular T helper (Tfh) cells. Tfh cells provide B‐cell help in the lymph nodes and express CXCR5, that is, a receptor used for homing to lymphoid tissues [3].

More recently, another B‐cell helper T‐cell subset, peripheral T helper (Tph) cells, has been described. These cells were first detected in the synovial fluid and tissue of patients with rheumatoid arthritis (RA) patients, and phenotypically resemble Tfh, in particular through the secretion of IL‐21, a central cytokine for B‐cell differentiation and CXCL13, a B‐cell chemoattractant [4]. In contrast to Tfh cells, they do not express CXCR5 but rather chemokine receptors, such as CCR2 and CCR5 that allow their homing to inflamed tissues. There, they are hypothesized to be involved in the formation of ectopic lymphoid structures (ELS) that sustain local inflammatory and antibody responses [5]. In the context of T1D, the formation of ELS has been described both in the NOD mouse model during the development of autoimmune diabetes [6], and more recently also in patients with T1D as well as AAb^+^ at‐risk individuals [7]. Therefore, in addition to Tfh cells, Tph cells can also be envisioned to participate in the T1D disease process [8].

Blood CXCR5^+^, in particular, CXCR5^+^PD‐1^hi^ICOS^+^ memory CD4^+^ T cells are widely considered to represent the circulating counterpart of Tfh cells [9]. Similarly, blood CXCR5^−^PD‐1^hi^ memory CD4^+^ T cells phenotypically and transcriptomically resemble tissue Tph cells [5]. Circulating Tfh (cTfh) and Tph (cTph) cells also share several phenotypic characteristics, and they have been suggested to have a common clonal origin, deriving from the clonal expansion of Tfh cells within the germinal centers [10]. Importantly, multiple studies have demonstrated that both cTfh as well as cTph frequencies are increased in patients with autoimmune diseases, such as RA, systemic lupus erythematosus (SLE), and celiac disease [4, 11, 12, 13, 14, 15, 16, 17, 18]. Previous studies by us and others have demonstrated that both cTfh cells and cTph cells are similarly expanded in patients with T1D [19, 20, 21, 22]. Interestingly, abatacept, a CTLA‐4 fusion protein widely used to treat patients with RA, reduces cTfh and cTph frequencies, further supporting the association of these blood T‐cell subsets with autoimmunity [23, 24].

Both in RA and T1D, a few studies suggest that cTph but not cTfh frequencies are higher in at‐risk individuals already before the manifestation of clinical disease [22, 25]. However, the exact timing of the increase in the cTph frequency, that is, whether it is associated with the initiation or progression of autoimmunity, remains open. Moreover, it is currently unclear whether cTfh and cTph cells in patients with autoimmunity differ phenotypically from similar cells in healthy donors and whether the increased frequencies of cTfh and cTph cells are clonally related. In this study, we addressed these open research questions by performing single‐cell analyses of cTfh and cTph cells from blood samples of children at different stages of T1D progression.

## 2 Results

### 2.1 CXCR5^−^PD‐1^hi^ cTph Cells Are More Frequent but Phenotypically Similar in Children with Newly Diagnosed Type 1 Diabetes and in AAb^+^ Children Compared with Healthy Age‐Matched Controls

**FIGURE 1:**
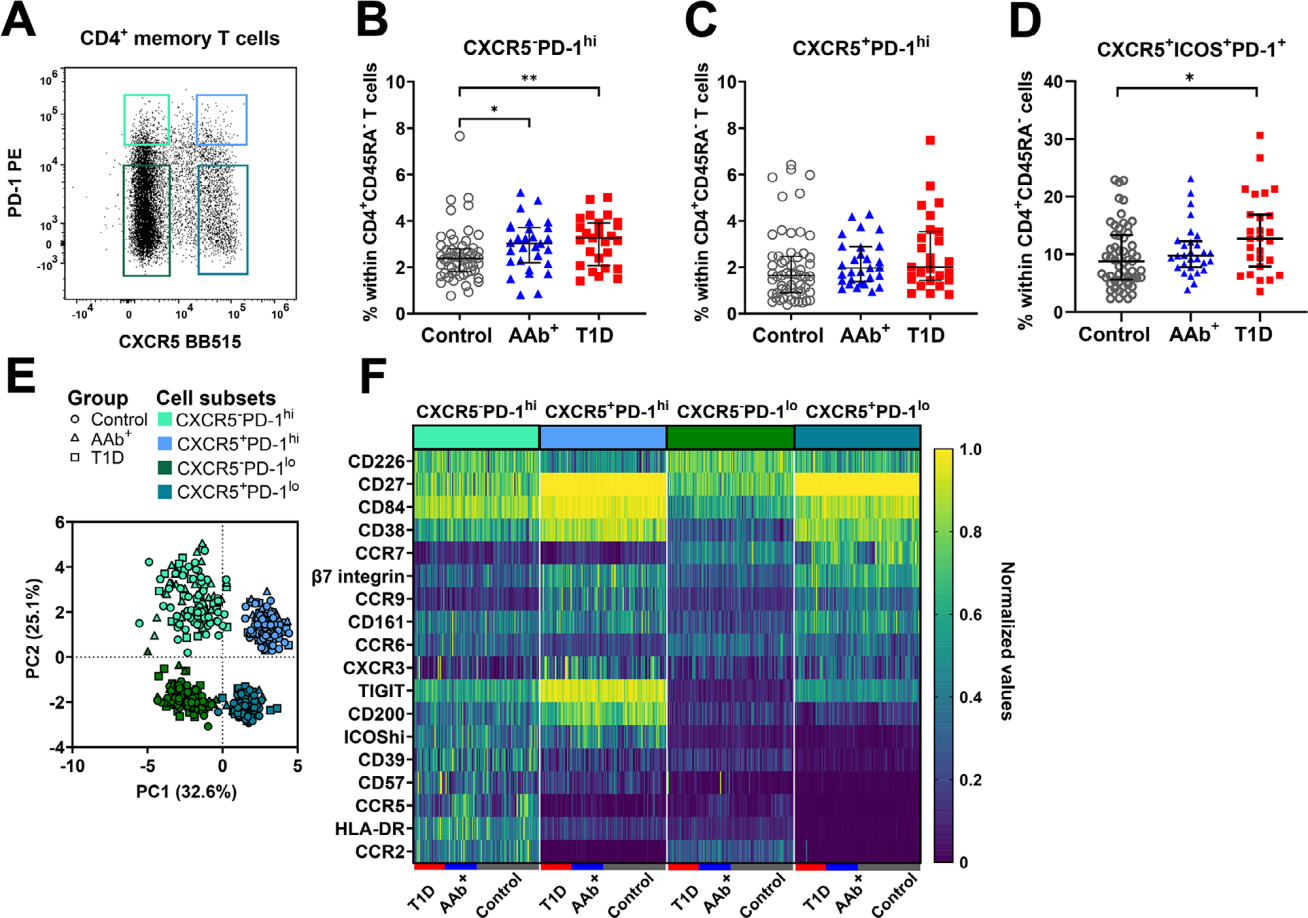
CXCR5^−^PD‐1^hi^ cTph cells are expanded in AAb^+^ children and children with newly diagnosed T1D compared with healthy controls. (A) Representative flow cytometry gating of viable memory T cells (CD4^+^CD45RA^−^) divided into four fractions according to CXCR5 and PD‐1 expression. Frequency of CXCR5^−^PD‐1^hi^ cTph (B), CXCR5^+^PD‐1^hi^ cTfh cells (C), and CXCR5^+^PD‐1^+^ICOS^+^ activated cTfh cells (D) within the pediatric cohort. (E) Dimensionality reduction analysis of the four CXCR5^+^/^−^PD‐1^hi^/^lo^ fractions using principal component analysis (PCA). (F) Heatmap of surface receptor expression within CXCR5^+^/^−^PD‐1^hi^/^lo^ fractions from children with T1D, AAb^+^ children, and controls. Kruskal–Wallis test with Dunn's multiple comparisons was used in the statistical analysis. For PCA and heatmap generation, the expression of each marker was normalized by giving the smallest percentage in each row a value of 0 and the highest percentage in each row a value of 1.0. The median and interquartile range (IQR) are shown in (B–D). *n* = 57 control; *n* = 29 AAb^+^; *n* = 27 T1D. *p < 0.05, **p < 0.01. Controls are depicted as open grey circles, AAb^+^ children as solid blue triangles, and children with newly diagnosed T1D as solid red squares in (B–D).

As previously described [4, 15, 22], based on CXCR5 and PD‐1 expression we divided peripheral blood memory CD4^+^ T cells into CXCR5^−^PD‐1^hi^ (putative cTph cells) and CXCR5^+^PD‐1^hi^ (putative cTfh cells), as well as into CXCR5^−^PD‐1^lo^ and CXCR5^+^PD‐1^lo^ fractions (Figure 1A), and analyzed these in a cohort including 27 children with newly diagnosed T1D, 29 AAb^+^ subjects who later progressed to T1D, as well as 57 age‐matched healthy controls. Analogous to our earlier analysis [22], we confirmed in this cohort that both children with newly diagnosed T1D and AAb^+^ children exhibit a higher frequency of CXCR5^−^PD‐1^hi^ cTph cells compared with healthy, age‐matched controls (Figure 1B), while the differences in the frequencies of CXCR5^+^PD‐1^hi^ cTfh cells were less pronounced (Figure 1C). However, again in line with our earlier studies [21] the frequency of “activated cTfh cells” (defined as CXCR5^+^PD‐1^+^ICOS^+^) was increased in children with T1D (Figure 1D; Figure S1). These results were confirmed by pairwise analysis of age‐matched sample pairs processed in parallel (Figure S1). Of note, the HLA class II genotype or the type of first positive AAb detected in AAb^+^ subjects did not influence cTph or cTfh cell frequencies (Figures S2 and S3, respectively). Moreover, no correlations between cTph and cTfh frequencies and clinical parameters (plasma glucose and β‐hydroxybutyrate levels, and blood pH at diagnosis) were observed in children with newly diagnosed T1D (Figure S4).

Next, we analyzed the expression of 18 surface markers reported to be differentially expressed by cTfh and cTph cells in previous studies [4, 15, 16, 22] on CXCR5^−^PD‐1^hi^ cTph, CXCR5^+^PD‐1^hi^ cTfh, as well as CXCR5^−^PD‐1^lo^ and CXCR5^+^PD‐1^lo^ cells from children with T1D, AAb^+^ children and healthy controls. In a principal component analysis (PCA), the cells are visually clustered according to the cell fractions rather than the study groups (Figure 1D). Of the 18 markers analyzed, five markers (CD84, CD38, TIGIT, CD200, and ICOS) were expressed at a significantly higher level, and one marker, CCR7, at a lower level in both CXCR5^−^PD‐1^hi^ cTph and CXCR5^+^PD‐1^hi^ cTfh fractions, compared with the corresponding PD‐1^lo^ fractions (Figure 1E; Tables S1 and S2). When comparing CXCR5^−^PD‐1^hi^ cTph and CXCR5^+^PD‐1^hi^ cTfh cells, cTph cells demonstrated a significantly higher expression of seven markers (CD226, CCR6, CD39, CD57, CCR5, HLA‐DR, and CCR2). Conversely, the expression of eight markers (CD27, CD84, CD38, β7 integrin, CCR9, CXCR3, TIGIT, and CD200) was higher on cTfh cells (Figure 1E; Table S3).

Next, we compared the expression of these 18 markers on CXCR5^−^PD‐1^hi^ cTph and CXCR5^+^PD‐1^hi^ cTfh cells between children with T1D and AAb^+^ children and healthy controls. As already suggested by the PCA projection, no differences were observed between the study groups (Figure S5–S8). In conclusion, our flow cytometry analyses demonstrate an increased frequency of cTph cells both in children with T1D and in AAb^+^ children who later progressed to T1D compared with controls. However, phenotypically these expanded cTph cells appear not to differ from the cTph cells of healthy children.

### 2.2 CXCR5^−^PD‐1^hi^ and CXCR5^+^PD‐1^hi^ CD4^+^ T Cells Share the Expression of Several Transcripts Associated with B‐Cell Helper Function

**FIGURE 2:**
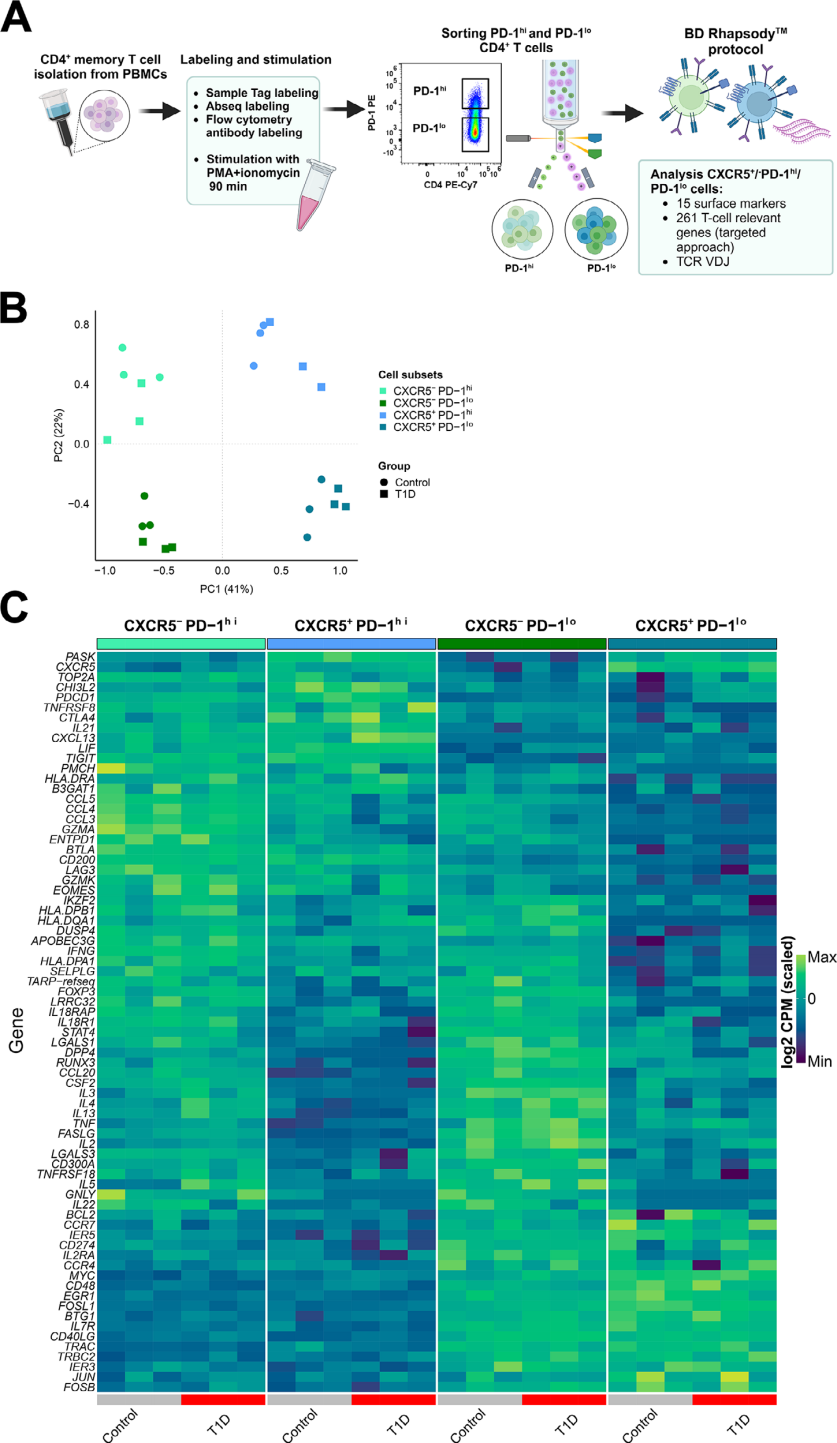
Both CXCR5^−^PD‐1^hi^ and CXCR5^+^PD‐1^hi^ fractions express transcripts associated with B‐cell helper T‐cell function. (A) Outline of the single‐cell multiomics workflow. (B) T cells were divided into four CXCR5^−^/^+^PD‐1^hi^/^lo^ fractions according to CXCR5 and PD‐1 expression. PCA plot based on all analyzed genes (196). (C) Heatmap of genes significantly (FDR <0.05) up‐ or downregulated between CXCR5^−^/^+^/PD‐1^hi^/^lo^ fractions from healthy controls and children with T1D. Panel A was created with Biorender.com.

To further validate our phenotypic analysis of CXCR5^−^PD‐1^hi^ cTph and CXCR5^+^PD‐1^hi^ cTfh cells, we next performed a targeted gene expression analysis in parallel with surface protein expression (AbSeq) and TCR repertoire analyses using the BD Rhapsody Single‐Cell Analysis System. For this experiment, memory CD4^+^ T cells from three children with newly diagnosed T1D and three age‐matched healthy controls were briefly stimulated with PMA and ionomycin to enable the detection of cytokine transcripts [26, 27], sorted into PD‐1^hi^ and PD‐1^lo^ fractions, and subsequently pooled at a 1:1 ratio to enrich for the rare PD‐1^hi^ cells for the single‐cell analyses (Figure 2A). CXCR5^−^/^+^ T cells were selected for analysis based on CXCR5 protein expression (Figure S9).

First, we compared the CXCR5^−^/^+^PD‐1^hi^/^lo^ fractions through a pseudobulk differential expression analysis, with the PCA results mirroring those obtained in flow cytometry analyses (Figure 2B). Both CXCR5^−^PD‐1^hi^ cTph and CXCR5^+^PD‐1^hi^ cTfh fractions expressed *IL21*, *CXCL13*, *TIGIT*, *CCL4*, *CCL3*, *CD200*, *GZMK*, and *EOMES* at significantly higher levels compared with the corresponding PD‐1^lo^ fractions (Figure 2C; Tables S4 and S5). When comparing CXCR5^−^PD‐1^hi^ cTph and CXCR5^+^PD‐1^hi^ cTfh fractions, the cTph fraction demonstrated a significantly higher expression of genes encoding inflammatory chemokines and cytokines, such as *CCL4, CCL3, CCL20, IFNG, CSF2, IL13*, and *IL22*, as well as cytotoxic molecules, such as *GZMA* and *GNLY*. (Figure 2C; Table S6). Gene ontology (GO) enrichment analysis also revealed notable differences between the four different fractions (Table S7). However, no differences between the limited number of samples from children with T1D and healthy controls were observed in the pseudobulk analyses, with the exception of upregulated *IL5* expression in CXCR5^−^PD‐1^hi^ cTph fraction of children with T1D (Figure 2C; Table S8).

To summarize, the pseudobulk analysis of single‐cell multiomics data demonstrated that CXCR5^−^PD‐1^hi^ cTph and CXCR5^+^PD‐1^hi^ cTfh fractions share the expression of several transcripts associated with B‐cell helper function, in particular *CXCL13* and *IL21*, while cTph cells express higher levels of inflammatory chemokines and cytokines, as well as markers associated with cytotoxic function compared with cTfh cells.

### 2.3 TCR Sharing with CXCR5^−^PD‐1^hi^ cTph Cells Is Observed both Within the CXCR5^+^PD‐1^hi^ cTfh and CXCR5^−^PD‐1^lo^ CD4^+^ T Cells

**FIGURE 3:**
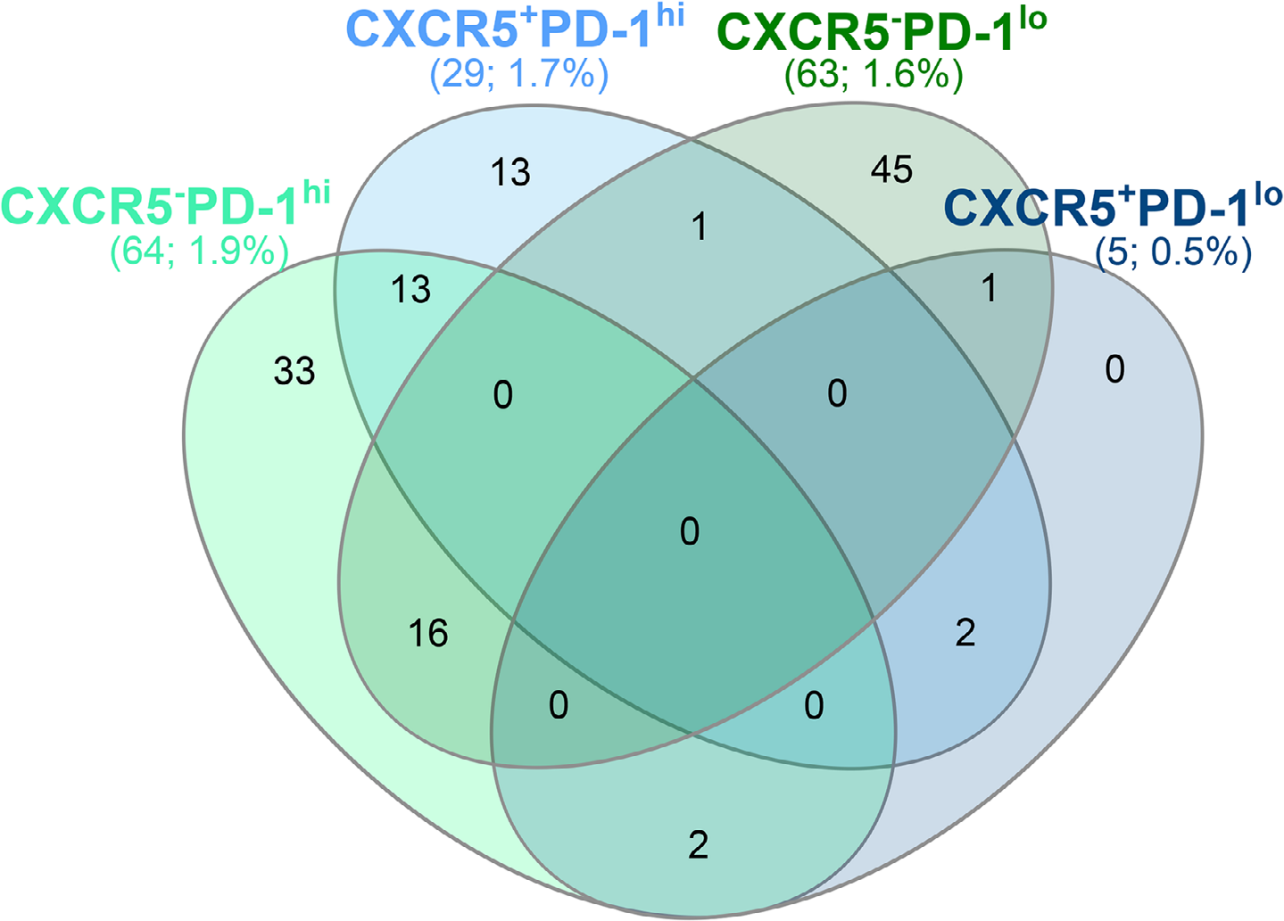
TCR sharing is most commonly observed between CXCR5^−^PD‐1^hi^ and CXCR5^−^PD‐1^lo^, or CXCR5^−^PD‐1^hi^ and CXCR5^+^PD‐1^hi^ CD4^+^ T cells. TCR sharing between CXCR5^−^/^+^PD‐1^hi^/^lo^ fractions. The total number and percentage of expanded clones detected within each cell fraction are indicated in parentheses. The number of TCR sequences detected within each fraction was: CXCR5^−^PD‐1^hi^, 3324; CXCR5^+^PD‐1^hi^, 1675; CXCR5^−^PD‐1^lo^, 3903; CXCR5^+^PD‐1^lo^, 853.

When analyzing the TCR repertoire within our single‐cell multiomics dataset, we detected 126 expanded clones (defined as ≥ 2 similar TCRs detected in individual T cells). All clones were private, that is, no clonal sharing was observed between different individuals. (Table S9). TCR sharing between the different CXCR5^−^/^+^PD‐1^hi^/^lo^ fractions occurred most prominently between the CXCR5^−^PD‐1^hi^ and CXCR5^−^PD‐1^1o^ fractions (16 clones) and between CXCR5^−^PD‐1^hi^ and CXCR5^+^PD‐1^hi^ fractions (13 clones; Figure 3). In summary, the TCR sequencing data suggest that CXCR5^−^PD‐1^hi^ cTph cells may be clonally related both with CXCR5^+^PD‐1^hi^ cTfh and CXCR5^−^PD‐1^1o^ cells.

### 2.4 Single‐Cell Multiomics Analysis Suggests Higher Phenotypic Heterogeneity Within CXCR5^−^PD‐1^hi^ CD4^+^ T Cells Compared with CXCR5^+^PD‐1^hi^ CD4^+^ T Cells

**FIGURE 4:**
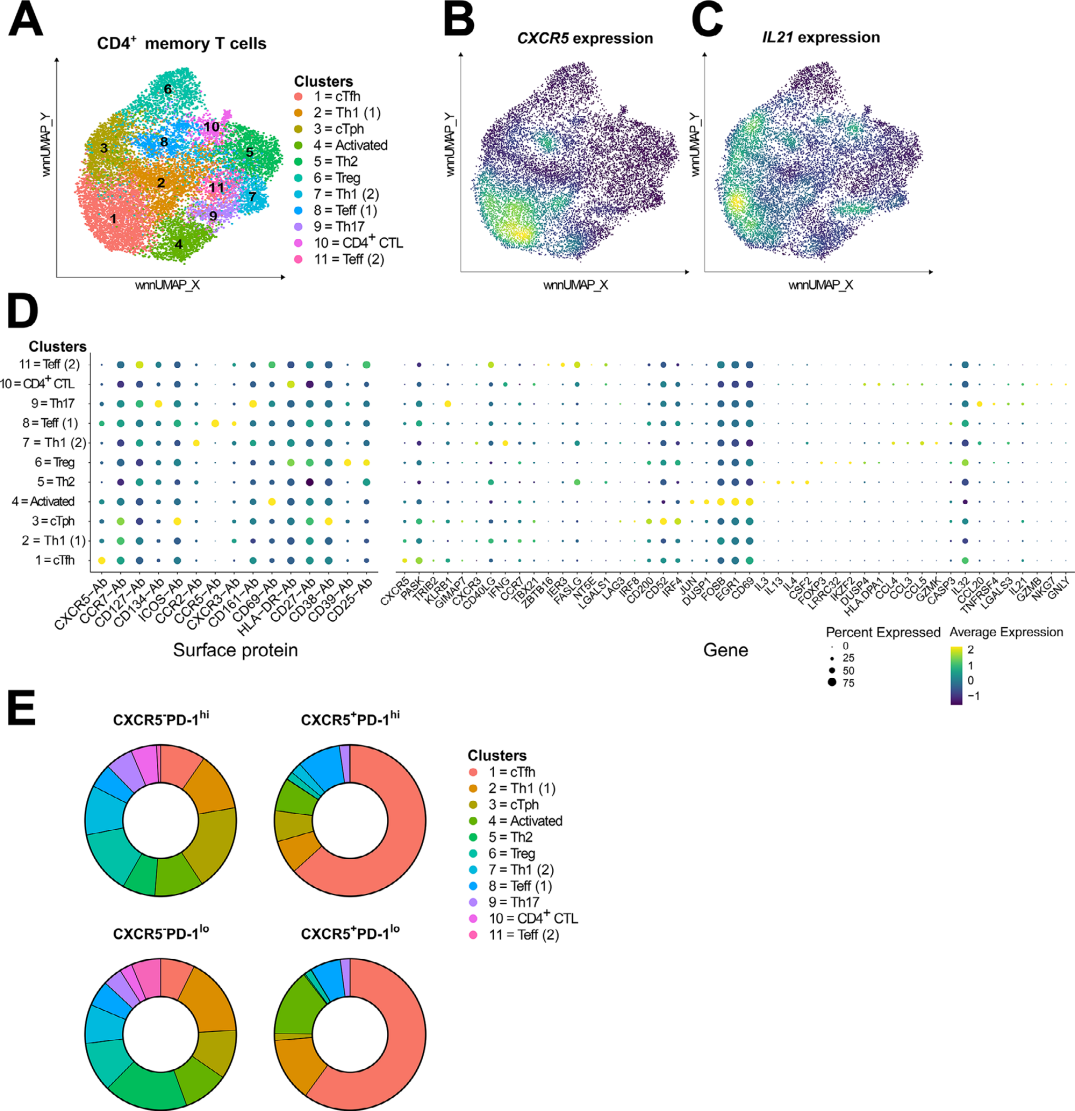
CXCR5^−^PD‐1^hi^ CD4^+^ T cell fraction contains multiple effector cell types. (A) WNN UMAP projection of the 11 identified T‐cell clusters. Expression of CXCR5 protein (B) and *IL21* gene (C) within WNN UMAP. (D) Dotplot of surface proteins and genes differentially expressed within the cell clusters. The top 5 unique, significantly differentially expressed genes per cluster were selected for the visualization. (E) The distribution of cells from each cluster in the four CXCR5^+^/^−^PD‐1^hi^/^lo^ cell fractions is depicted as pie charts. Differentially expressed proteins and genes per cluster were determined in the Seurat WNN pipeline using the Wilcoxon rank sum test in (D).

Finally, we performed unsupervised clustering and differential gene and surface protein expression analysis encompassing the whole dataset (CXCR5^+^/^−^PD‐1^hi^/^lo^ memory CD4^+^ T cells). We identified clusters representing major T‐helper cell populations, such as Th1, Th2, and Th17 cells and Tregs with this approach (clusters 1–11; Figure 4A; Tables S10 and S11). Among these clusters, cluster 1 appeared to represent cTfh cells, as indicated by the upregulation of CXCR5, *ICOS*, and *IL21*, and cluster 3 cTph cells, as indicated by the upregulation of *IL21, PDCD1*, ICOS, and CD38, and low expression of CXCR5 (Figure 4B–D). When the frequencies of these clusters within the four CXCR5^+^/^−^PD‐1^hi^/^lo^ fractions were analyzed, most of the cells within the CXCR5^+^PD‐1^hi^ fraction belonged to cluster 1 (cTfh), as expected. In contrast, the composition of the CXCR5^−^PD‐1^hi^ fraction was far more diverse. In addition to containing cells from cluster 3 (cTph), the CXCR5^−^PD‐1^hi^ fraction contained cells from multiple other clusters. In particular, cells from cluster 6, representing Tregs as indicated by the upregulation of *FOXP3*, cluster 7, representing Th1‐like proinflammatory cells as indicated by the upregulation of *CXCR3*, *IFNG*, and *GZMK*, and cluster 10, representing cytotoxic CD4^+^ T cells (CTLs), as indicated by the upregulation of *GZMB, NKG7* and *GNLY*, were enriched within the CXCR5^−^PD‐1^hi^ fraction compared with the other fractions. (Figure 4E; Figure S10). Consistent with this, clonal expansions observed within the CXCR5^−^PD‐1^hi^ fraction (Figure 3) appeared to be distributed across multiple clusters, including cluster 3 (Table S9).

In summary, single‐cell clusters with cTph and cTfh characteristics are heavily localized within the CXCR5^−^PD‐1^hi^ and CXCR5^+^PD‐1^hi^ fractions, respectively. However, in contrast to the highly homogeneous nature of the CXCR5^+^PD‐1^hi^ fraction, the CXCR5^−^PD‐1^hi^ fraction appeared more heterogeneous at the single‐cell level, containing T cells of diverse phenotypes in addition to those displaying cTph phenotype.

### 2.5 Comparable Frequencies of CXCR5^−^PD‐1^hi^ cTph Cells in Children During the Seroconversion to Islet Autoantibody Positivity

**FIGURE 5:**
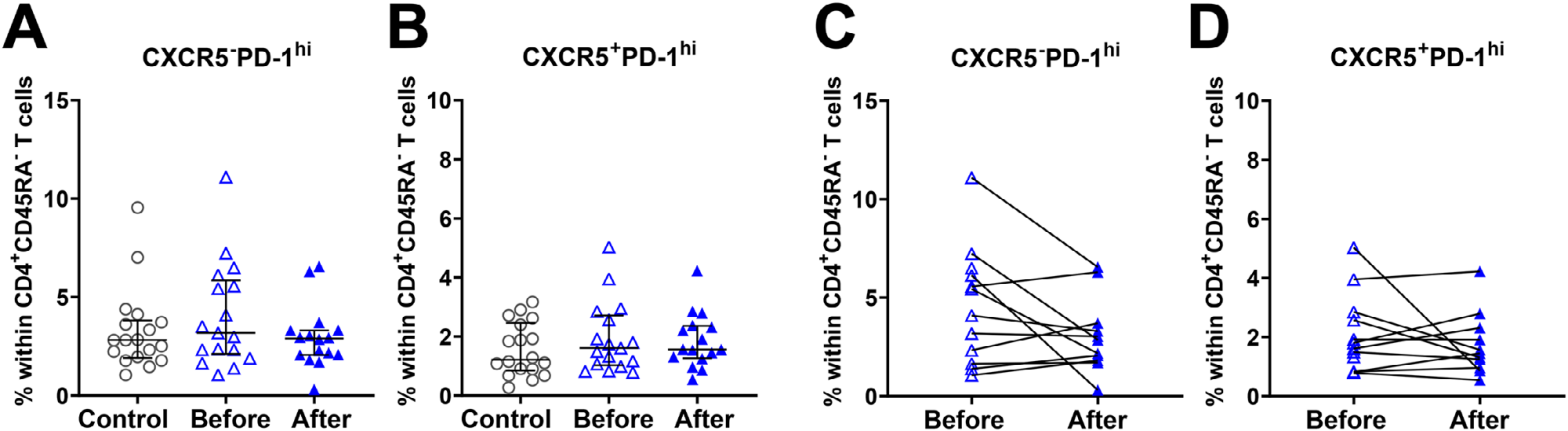
Comparable frequency of cTph and cTfh cells detected in samples collected before and after seroconversion to T1D‐associated autoantibodies. Frequency of CXCR5^−^PD‐1^hi^ cTph (A) and CXCR5^+^PD‐1^hi^ cTfh cells (B) in children before and after seroconversion for AAb‐positivity (*n* = 18 control; *n* = 17 before; n = 16 after). Pairwise analysis of frequencies of CXCR5^−^PD‐1^hi^ cTph (C) and CXCR5^+^PD‐1^hi^ cTfh (D) before and after seroconversion in children who later progressed to T1D (*n* = 11). Kruskal–Wallis with Dunn's multiple comparison test was used for statistical testing in analyses containing more than two groups. Wilcoxon matched‐pairs signed‐rank test was used for statistical analysis when two groups were compared. Median with IQR is indicated within each study group. Controls are depicted as open grey circles, children before seroconversion as open blue triangles, and children after seroconversion (AAb^+^) as solid blue triangles.

A major outstanding question is whether the increase in CXCR5^−^PD‐1^hi^ cTph cells occurs already during the early phases of autoimmunity or whether it is rather a feature of disease progression. To address this, we analyzed a rare longitudinal sample set from 17 younger children, with blood samples collected 3 to 13 months apart before and after the children turned seropositive for islet autoantibodies, an early time point thought to reflect the initiation of autoimmunity. Within this sample set, we observed a comparable frequency of CXCR5^−^PD‐1^hi^ cTph, CXCR5^+^PD‐1^hi^ cTfh, and CXCR5^+^PD‐1^+^ICOS^+^ activated cTfh before and after seroconversion, as well as compared with healthy age‐matched controls (Figure 5A,B; Figure S11). Since not all seroconverted children in the cohort had later progressed to T1D, we also performed a separate pairwise analysis of children who later progressed to clinical T1D (*n* = 11), with similar results (Figure 5C–D). Of note, we also studied the frequencies of different B‐cell subsets and T regulatory cells (Tregs) within this cohort, with no detectable differences between the samples. (Figures S12 and S13).

Taken together, in contrast to samples from AAb^+^ children collected closer to T1D diagnosis we did not detect a clear increase in the frequency of CXCR5^−^PD‐1^hi^ cTph cells at seroconversion, an earlier stage of the autoimmune process, suggesting that the increase in the frequency of cTph cells occurs closer to the progression to clinical T1D.

## 3 Discussion

In this study, we performed an extensive analysis of CXCR5^−^PD‐1^hi^ cTph and CXCR5^+^PD‐1^hi^ cTfh cells at the single‐cell level to elucidate outstanding questions on the timing of their emergence during T1D autoimmunity, cellular heterogeneity, and relationship with each other. Reassuringly, we first confirmed our previous observations that the frequencies of both cTph and activated cTfh cells were increased in children with newly diagnosed T1D [21, 22]. In addition, we validated our previous finding that cTph but not cTfh frequencies are expanded already before the onset of symptomatic disease in AAb^+^ children who later progressed to T1D [22].

To further evaluate when the cTph expansion occurs during autoimmunity, we utilized rare samples from younger children collected before and after their initial seroconversion, an early time point thought to reflect the onset of autoimmunity. However, no alterations in the frequencies of cTph, or cTfh cells were observed during seroconversion in this cohort. Our current results support the idea that the increase in the frequency of cTph cells is a phenomenon associated with later progression to clinical T1D rather than initiation of autoimmunity and/or the emergence of autoantibodies. This notion is also supported by our earlier observation that cTph frequencies were only increased in AAb^+^ children who later progressed to T1D but not in those who did not progress [22]. Highly similar observations have recently been made in the context of another autoimmune disease, RA, where cTph, but again not cTfh, frequencies were increased in at‐risk individuals prior to the manifestation of clinical disease [25]. Importantly, cTph frequencies have also been shown to correlate with disease activity in patients with SLE [15] and RA [4, 17], and to decrease upon immunomodulatory treatment [17, 23, 24]. Taken together, since cTph frequencies appear to be increased during active autoimmunity, they can be speculated to reflect ongoing extrafollicular B cell‐T cell interactions in ELSs, such as those recently observed in the pancreases of patients with T1D or AAb^+^ individuals at‐risk for T1D [7].

In accordance with previous studies [4, 15, 22], we demonstrate here that cTph and cTfh cells share the expression of several markers associated with B‐cell helper function, such as IL‐21, CXCL13, ICOS, and TIGIT, at the transcript and/or protein level, while CCR2 and CCR5, chemokine receptors associated with homing to inflamed tissues, are more abundantly expressed by cTph cells. However, neither our flow cytometry nor single‐cell multiomics analyses revealed any major phenotypic differences between cTph or cTfh cells from children with T1D compared with controls. In contrast, increased expression of Tph markers, such as CXCL13, ICOS, and TIGIT has been reported in cTph cells from patients with SLE and RA compared with cTph cells from healthy controls [15, 25]. This could reflect a more prominent Tph activation in these diseases compared with T1D, which is also supported by the finding that the increases in cTph frequencies appear to be higher in SLE and RA compared with T1D [4, 5, 15, 25].

Our single‐cell multiomics analysis revealed also interesting differences between CXCR5^−^PD‐1^hi^ and CXCR5^+^PD‐1^hi^ fractions. Namely, while the CXCR5^+^PD‐1^hi^ fraction contained a homogeneous population of cells with a cTfh phenotype, the CXCR5^−^PD‐1^hi^ fraction, in contrast, appeared considerably more heterogeneous. In addition to cells expressing a prototypical cTph signature, CXCR5^−^PD‐1^hi^ cells appeared to contain cells carrying, for example, Treg, Th1, or CTL characteristics. The heterogeneity of blood CXCR5^−^PD‐1^hi^ CD4^+^ T cells has also been demonstrated in previous studies. For example, Caielli et al. [28] reported that SLE patients harbor an increased frequency of circulating CXCR5^−^PD‐1^hi^ CXCR3^+^ CD4^+^ T cells that express IL‐10, but not IL‐21 or CXCL13. More recently, Goto et al. [29] identified a population of CXCR3^mid^ CD4^+^ T cells, which they coined “age‐associated T‐helper cells” (ThA). These ThA cells appeared to constitute a subset of CXCR5^−^PD‐1^hi^ CD4^+^ T cells, and in addition to expressing IL‐21 and CXCL13, they also expressed granzymes A and B, and perforin, cytotoxic molecules characteristic of CD4^+^ CTLs [29]. Finally, Elahee et al. [30] recently demonstrated that CXCR5^−^PD1^hi^HLA‐DR^+^ICOS^−^ but not total CXCR5^−^PD1^hi^ CD4^+^ T cells were expanded in patients with systemic sclerosis and that these cells appeared to express cytotoxic rather than B‐cell helper features [30]. Together with our current results, these observations suggest that the CXCR5^−^PD‐1^hi^ definition may also encompass cell types distinct from cTph cells within blood CD4^+^ T cells. Therefore, the expansions of cTph cells (defined as CXCR5^−^PD‐1^hi^ CD4^+^ T cells) reported in patients with autoimmune diseases could potentially reflect a more heterogeneous T‐cell response. However, as already noted above, in this study we did not detect any prominent phenotypic subpopulation within CXCR5^−^PD‐1^hi^ CD4^+^ T cells in patients with T1D, at least not based on the 18 phenotypic markers analyzed.

The seminal study by Del Alcazar et al. [10] suggested through an in‐depth analysis of blood and lymph node T cells that cTfh and cTph may share a common clonal origin, deriving from the clonal expansion of germinal center Tfh cells. The TCR repertoire analysis of our single‐cell multiomics data set also supports this notion, as a considerable clonal overlap was observed between cTfh and cTph cells. cTph, but not cTfh cells, also demonstrated considerable clonal sharing with PD‐1^lo^ cells, a phenomenon most likely associated with the phenotypic heterogeneity of the cTph cells discussed above. However, due to the limited number of single T cells analyzed here, these analyses would need to be confirmed in a larger cohort in the future.

In summary, our current results suggest that the increased frequency of cTph cells in blood is a phenomenon likely associated with later progression to clinical T1D rather than the initiation of autoimmunity. Moreover, in both children with T1D as well as in AAb^+^ at‐risk subjects later progressing to T1D cTph cells appeared not to phenotypically differ from cTph cells of healthy control children, suggesting that the alteration of cTph cells in the context of T1D autoimmunity is rather quantitative than qualitative in nature. Finally, despite clonal and phenotypic similarities between cTph and cTfh cells, cTph cells (at least when defined as CXCR5^−^PD‐1^hi^ CD4^+^ T cells) appear more heterogeneous than cTfh cells at the single‐cell level, a finding that may need to be taken into account when using cTph cells as surrogate markers of extrafollicular B‐cell helper T cell activity.

### 3.1 Data Limitations and Perspectives

An obvious caveat of the current study is that the analyses were performed using circulating polyclonal CD4^+^ T cells, which may not be reflective of the phenotype of autoreactive CD4^+^ T cells. Studies on the phenotype of autoreactive CD4^+^ T cells in the context of T1D are limited, due to the difficulty of reliably detecting these rare cells in the circulation [31]. However, in two other autoimmune diseases, celiac disease [16] and autoimmune hepatitis [32], rare autoreactive CD4^+^ T cells have been shown to exhibit a phenotype largely consistent with the cTph signature, suggesting that polyclonal cTph expansions may also reflect the phenotype of autoreactive CD4^+^ T cells. Another potential caveat is the relatively small size of the seroconversion cohort, which may have reduced the statistical power to detect potential subtle changes in cTph, or cTfh, cells during the initiation of autoimmunity. Although difficult to obtain, a larger longitudinal sample series would be required to confirm our observations on the exact timing of cTph alterations during T1D progression. Lastly, the brief ex vivo PMA and ionomycin stimulation employed to enhance cytokine transcript detection might influence the transcriptome of the T cells. However, prior comparisons between unstimulated and stimulated T cells indicate that this short stimulation minimally affects the expression of other transcripts and does not distort the data [26, 27].

## 4 Materials and Methods

### 4.1 Study Subjects

The project analyzed two pediatric cohorts summarized in Tables S12 and S13. Cohort 1 consisted of 27 children with newly diagnosed T1D (0–7 days after clinical diagnosis), 29 AAb^+^ children, who later progressed to clinical T1D (0.5–3.9 years after sampling), and 57 healthy control children (Table S1). The control children were autoantibody‐negative and age‐matched with T1D and AAb^+^ children. Cohort 2 consisted of samples from 17 children collected before and after seroconversion for T1D‐associated autoantibodies, and 18 age‐matched autoantibody‐negative control children (Table S2). The AAb^+^ children and healthy control children participated in the Finnish Type 1 Diabetes Prediction and Prevention (DIPP) follow‐up study. Donors participating in the DIPP study have HLA class II genotypes associated with increased risk for T1D. Autoantibody positivity was analyzed as previously described [33]. Autoantibody‐positivity was defined based on positivity for one or more biochemical autoantibodies (IAA, IA‐2A, and GADA). The blood samples were collected between March 2009 and January 2019. Sex was registered for each study subject, but it was not considered a factor in the statistical analysis.

### 4.2 Sample Preparation

PBMCs were isolated from heparinized peripheral blood samples using Ficoll gradient centrifugation and stored in liquid nitrogen until analyses.

### 4.3 Flow Cytometry

PBMCs were thawed, treated with DNAase I (StemCell), and rested for 2 h at +37°C, 5% CO_2_ in RPMI‐1640 culture medium (Lonza) supplemented with 2 mM L‐glutamine (Lonza), 20 mM 2‐ME (Sigma), 1 mM sodium pyruvate (Lonza), nonessential amino acids (Lonza), 100 IU/mL penicillin (Lonza), 100 mg/mL streptomycin (Lonza), 10 mM HEPES (Lonza) and inactivated 5% human AB serum (Sigma). Immunostainings were performed with 0.3–0.5 × 10^6^ PBMCs per staining. First, the cells were labeled with the viability stain Zombie Aqua (Biolegend) according to the manufacturer's instructions (dilution 1:800). The cells were subsequently labeled with fluorochrome‐labeled antibodies (Table S14) for 30 min at room temperature. Brilliant Buffer Plus (BD Biosciences) was added to immunostainings containing more than one Brilliant Violet or Brilliant Blue dye according to the manufacturer's instructions. Fluorescence minus one controls were used where applicable. Samples were acquired on a Novocyte Quanteon flow cytometer (Agilent), and the data were analyzed using FlowJo version 10.7.1 (BD Biosciences) (Figure S14). All flow cytometry assays and analyses were performed in accordance with the “Guidelines for the use of flow cytometry and cell sorting in immunological studies” [34].

### 4.4 Preparation of PBMCs for Single‐Cell Multiomics Analysis with BD Rhapsody Platform

Frozen PBMC samples from three children with newly diagnosed T1D and three healthy age‐matched controls were thawed and rested overnight at +37°C, 5% CO_2_. CD4^+^ memory T cells were isolated using the Memory CD4+ T cell Isolation Kit (Miltenyi). Cells were subsequently labeled with fluorochrome‐conjugated antibodies (Table S3), oligo‐nucleotide‐conjugated monoclonal AbSeq antibodies (Table S3), and with the Single Cell Sample Multiplexing kit (BD Biosciences). Cells were then stimulated for 90 min with phorbol myristate acetate (PMA, 50 ng/mL, Sigma) and ionomycin (1 µg/mL, Sigma) to enable the detection of cytokine transcripts [26, 27]. Finally, the cells were stained with the viability stain 7‐AAD (Biolegend) according to the manufacturer's instructions.

Viable CD4^+^CD45RA^−^PD‐1^hi^ and CD4^+^CD45RA^−^PD‐1^lo^ T cells were sorted from each donor with the Sony MA900 cell sorter (Sony Biotechnology, Figure S14), and samples from different donors were pooled into PD‐1^hi^ and PD‐1^lo^ fractions in Rhapsody sample buffer (BD Biosciences) (Figure 2A).

### 4.5 Library Preparation

Single cells were captured using the BD Rhapsody Express Single Cell Analysis (BD Biosciences) system and libraries were prepared separately for PD‐1^hi^ and PD‐1^lo^ samples according to manufacturer's protocols for mRNA expression of 261 genes (259 genes from Targeted T cell Expression Panel human, BD Biosciences and two supplemental genes, BD Biosciences, Table S15), for the expression of 15 cell surface receptors (AbSeq antibody‐oligonucleotide conjugates, BD Biosciences, Table S3), for the Sample Tags (BD single cell sample multiplexing kit) to perform multiplexing, and for TCR CDR3s to perform VDJ analysis. For additional details on library preparation, sequencing, and quality control, see the Supporting Information.

### 4.6 Single‐Cell Multiomics Data Analysis

Data analysis was carried out in R [35] using Seurat version 4.1.3 [36]. For technical details, see the Supporting Information. Clusters were annotated manually according to the gene and protein expression, as well as known cell‐type specific marker genes in each cluster. Seurat [36] and Nebulosa [37] packages were used for the visualization of dot plots and the expression of selected markers. Differentially expressed genes and surface proteins for each cluster were determined using the Wilcoxon rank sum test implemented in the FindAllMarkers function in Seurat. Genes and surface proteins were considered significantly differentially expressed when the log2‐fold change was ±0.25, and the Bonferroni adjusted *p*‐value ≤ 0.05.

### 4.7 Pseudobulk Analysis

Pseudobulk analysis was carried out for CXCR5^−^PD‐1^hi^, CXCR5^+^PD‐1^hi^, CXCR5^−^PD‐1^lo^, and CXCR5^+^PD‐1^lo^ cell fractions, that is, the fractions mirroring those analyzed by the flow cytometry approach. Pseudobulk analysis was carried out using edgeR [38, 39, 40, 41] version 3.40.0. For technical details, see the Supporting Information. Genes with FDR value ≤ 0.05 were considered significantly differentially expressed. Heatmap was created using the ComplexHeatmap package [42].

### 4.8 TCR Analysis

The TCR analysis was performed on 9900 T cells with paired TCRα and TCRβ chain sequences. Clones were defined as single T cells expressing identical TRAV, TRAJ, TRA CDR3, TRBV, TRBJ, and TRB CDR3 sequences. Venn diagram was created using InteractiVenn [43].

### 4.9 Statistics

Statistical analyses for flow cytometry data were performed using Prism version 9.1.0 (GraphPad). Statistical comparisons of >2 groups were performed using the Kruskal–Wallis or Friedmann test with Dunn's multiple comparison tests, as indicated in the figure legends and/or the tables. When two groups were compared, the Mann–Whitney *U* or Wilcoxon matched‐pairs signed rank test was used, as indicated in the figure legends. Associations between cell frequencies and different clinical variables were examined using Spearman's correlation. Statistical analysis of multiomics data was carried out with data type‐specific statical methodology as outlined above. *p *< 0.05 was considered to indicate statistical significance. No power calculations were made as the study was exploratory in nature with very limited previous data to support the calculations, and sample sizes were determined in part by feasibility.

## Author Contributions

**Anna‐Mari Schroderus**: Methodology, investigation, formal analysis, visualization, writing – original draft, writing – review & editing. **Andrea Hanel**: Formal analysis, writing – review & editing. **Céline Vandamme**: Methodology, writing – review & editing. **Viola Pitkänen**: Investigation, formal analysis, writing – review & editing. **Marja Rytkönen‐Nissinen**: Investigation, writing – review & editing. **Merja Heinäniemi**: Formal analysis, writing – review & editing. **Jorma Toppari**: Resources, writing – review & editing. **Mikael Knip**: Resources and writing – review & editing. **Riitta Veijola**: Resources and writing – review & editing. **Jorma Ilonen**: Resources and writing – review & editing. **Johanna Lempainen**: Resources and writing – review & editing. **Tuure Kinnunen**: Conceptualization, supervision, formal analysis, writing – original draft, writing – review & editing, and funding acquisition.

## Conflicts of Interest

The authors declare no conflicts of interest.

## Ethics Statement

All participants and/or their legal guardians have given written informed consent as mandated by the Declaration of Helsinki, and the privacy rights of human subjects have been followed. The study was approved by the local ethics committee at the Turku University Hospital (50/1801/2013; April 16, 2013), and the DIPP study by the ethics committee of the Hospital District of Northern Ostrobothnia (129/2003; December 15, 2003). All procedures were performed in compliance with relevant laws and institutional guidelines. Coded samples were used throughout the study.

## Peer Review

The peer review history for this article is available at https://publons.com/publon/10.1002/eji.202451704.

## Supporting information




**Supporting File 1**: eji6006‐sup‐0001‐SuppMat.pdf.

**Supporting File 2**: eji6006‐sup‐0002‐SuppMat.xlsx.

## Data Availability

The code used to analyze the single‐cell multiomics data is openly available on GitHub (https://github.com/annamsch665/cTph‐cTfh‐Schroderus‐Hanel). The datasets generated and/or analyzed during the current study are available from the corresponding author upon reasonable request.
