# Supplementary material for: The Frequency but not the Phenotype of Circulating Peripheral T Helper Cells is Increased at Later Stages of Progression to Type 1 Diabetes
Source: Eur J Immunol. 2025 Jun 20;55(6):e51704. doi: 10.1002/eji.202451704 (PMC12179578; doi:10.1002/eji.202451704)
Supplement: Supplementary file 1 — Supporting File 1: eji6006‐sup‐0001‐SuppMat.pdf. [file EJI-55-e51704-s001.pdf]

SUPPORTING INFORMATION

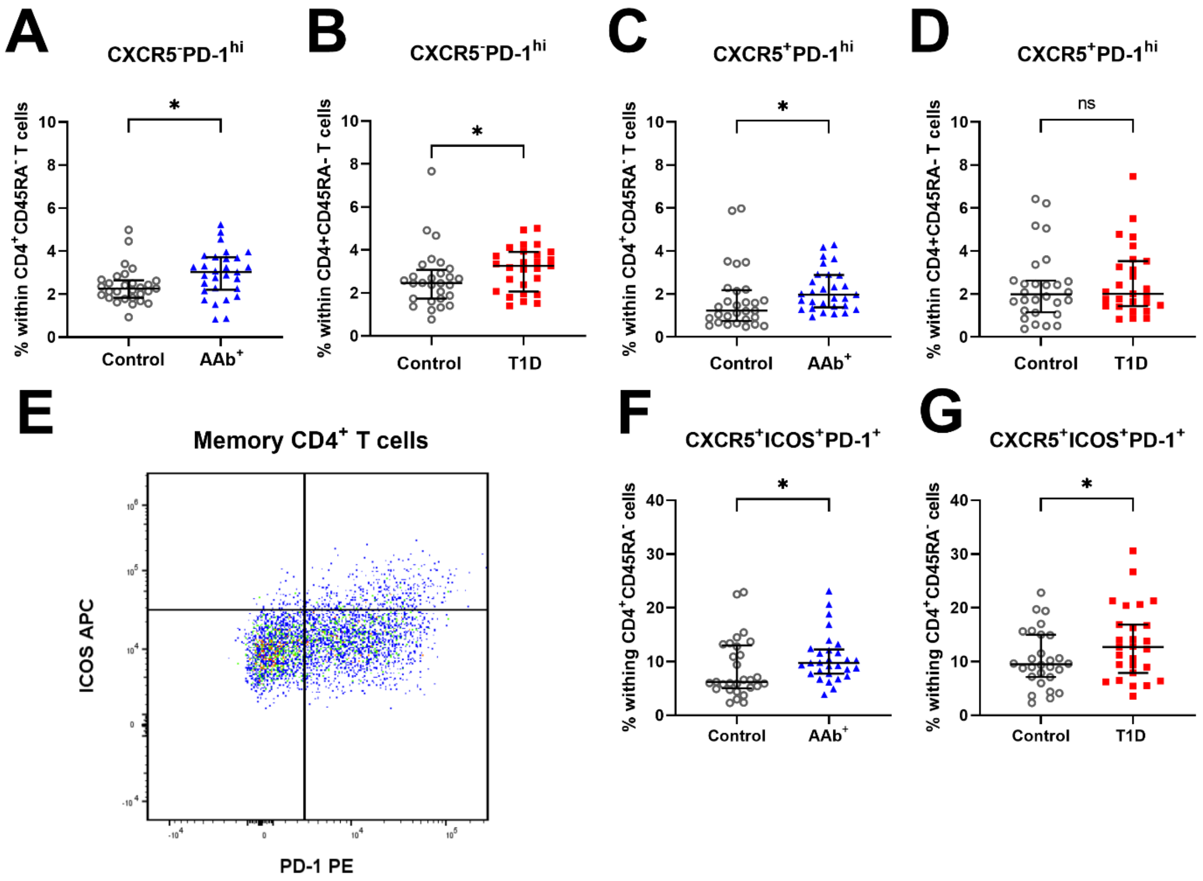

Supporting Figure 1. Pairwise analysis of CXCR5$^-$PD-1^{hi} cTph, CXCR5$^+$PD-1^{hi} cTfh and CXCR5$^+$ICOS$^+$PD-1$^+$ cTfh frequencies. Frequency of CXCR5$^-$PD-1^{hi} cTph cells in AAb$^+$ children and healthy age-matched controls **(A)** ,and in children with newly diagnosed T1D and healthy age-matched controls **(B)**. Frequency of CXCR5$^+$PD-1^{hi} cTfh cells in AAb$^+$ children and healthy age-matched controls **(C)**, and in children with newly diagnosed T1D and healthy age-matched controls **(D)**. Representative figure of gating activated Tfh i.e. CXCR5$^+$ICOS$^+$PD-1$^+$ cTfh cells **(E)**. Pairwise analysis of frequency of activated CXCR5$^+$ICOS$^+$PD-1$^+$ cTfh cells in AAb$^+$ children and healthy age-matched controls **(F),** and in children with newly diagnosed T1D and healthy age-matched controls **(G)**. Wilcoxon matched-pairs signed rank test was used for statistical analysis in B-D and F-G to compare age-matched samples processed and stained in parallel. Median with IQR is shown in the figures. *P<0.05.

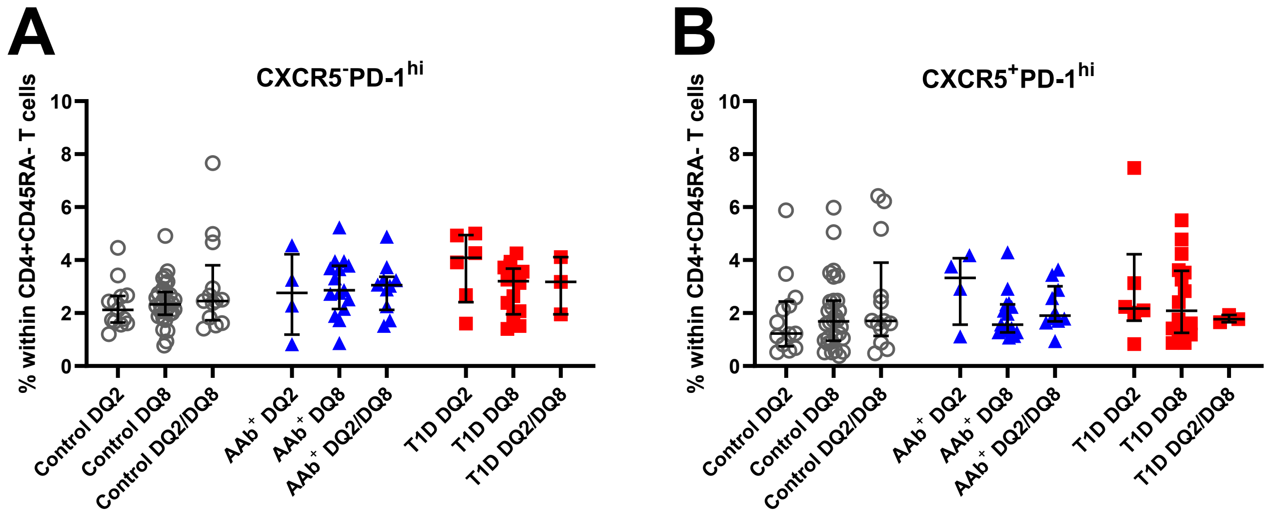

Supporting Figure 2. No effect by HLA class II genotype on cTph and cTfh frequencies. Frequencies of $CXCR5^{-}PD\text{-}1^{hi}$ cTph **(A)** and $CXCR5^{+}PD\text{-}1^{hi}$ cTfh cells **(B)** stratified based on the HLA class II genotype of the subjects. Kruskal-Wallis with Dunn's multiple comparison was used in statistical analysis comparing DQ2 (DQ2/DQ2 or DQ2/X), DQ8 (DQ8/DQ8 or DQ8/X) and DQ2/DQ8 groups within controls, AAb^{+} children or children with T1D. Median with IQR is shown in the figures. Controls are depicted as open grey circles, AAb^{+} children as solid blue triangles and children with newly diagnosed T1D as solid red squares.

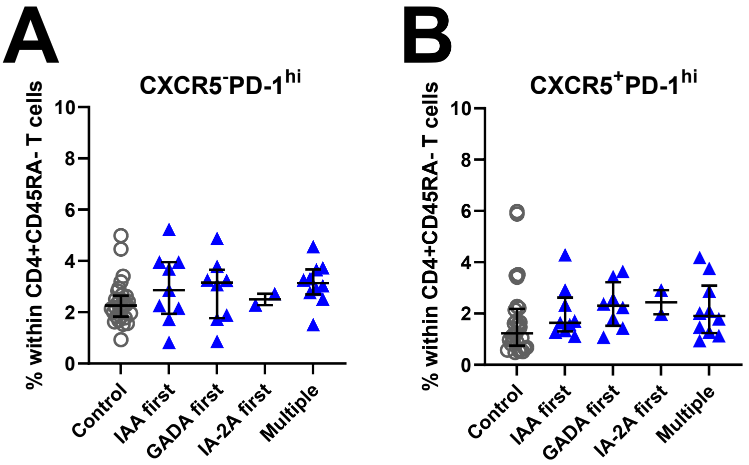

Supporting Figure 3. No effect by the first appearing autoantibody on the frequency of CXCR5$^-$PD-1^{hi} cTph and CXCR5$^+$PD-1^{hi} cTfh cells. Frequencies of CXCR5$^-$PD-1^{hi} cTph cells **(A)** and CXCR5$^+$PD-1^{hi} cTfh cells **(B)** in AAb$^+$ children. Children were divided into subgroups according to the first autoantibody detected during follow-up. Kruskal-Wallis with Dunn's multiple comparison test was used in statistical testing comparing the different subgroups of AAb$^+$ children. Median with IQR is shown in the figures.

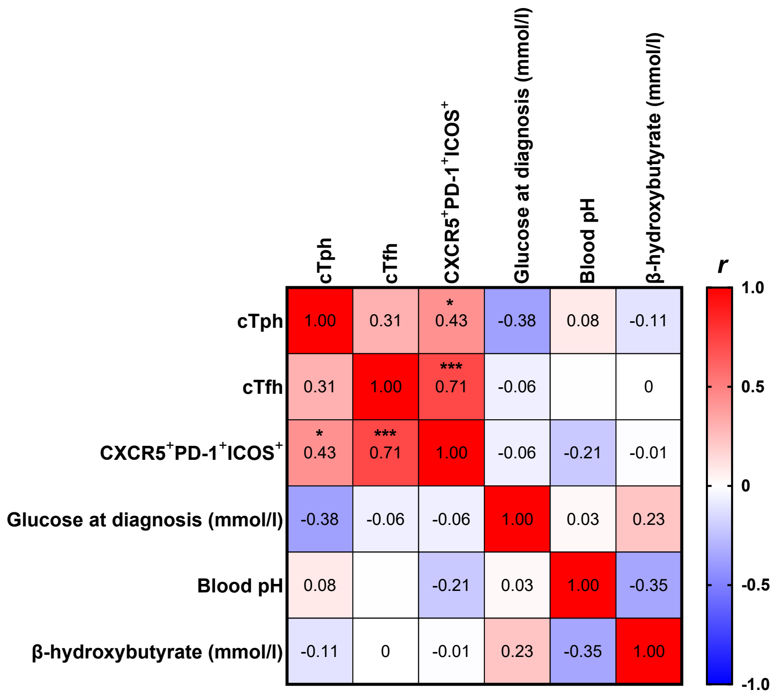

Supporting Figure 4. Correlation analysis between frequencies of CXCR5$^-$PD-1^{hi} cTph, CXCR5$^+$PD-1^{hi} cTfh and CXCR5$^+$ICOS$^+$PD-1$^+$ activated cTfh cells and clinical parameters in children with newly diagnosed T1D. Spearman's correlation test was used with *r*-values presented in the figure. n=15. *P≤0.05, ***P<0.001

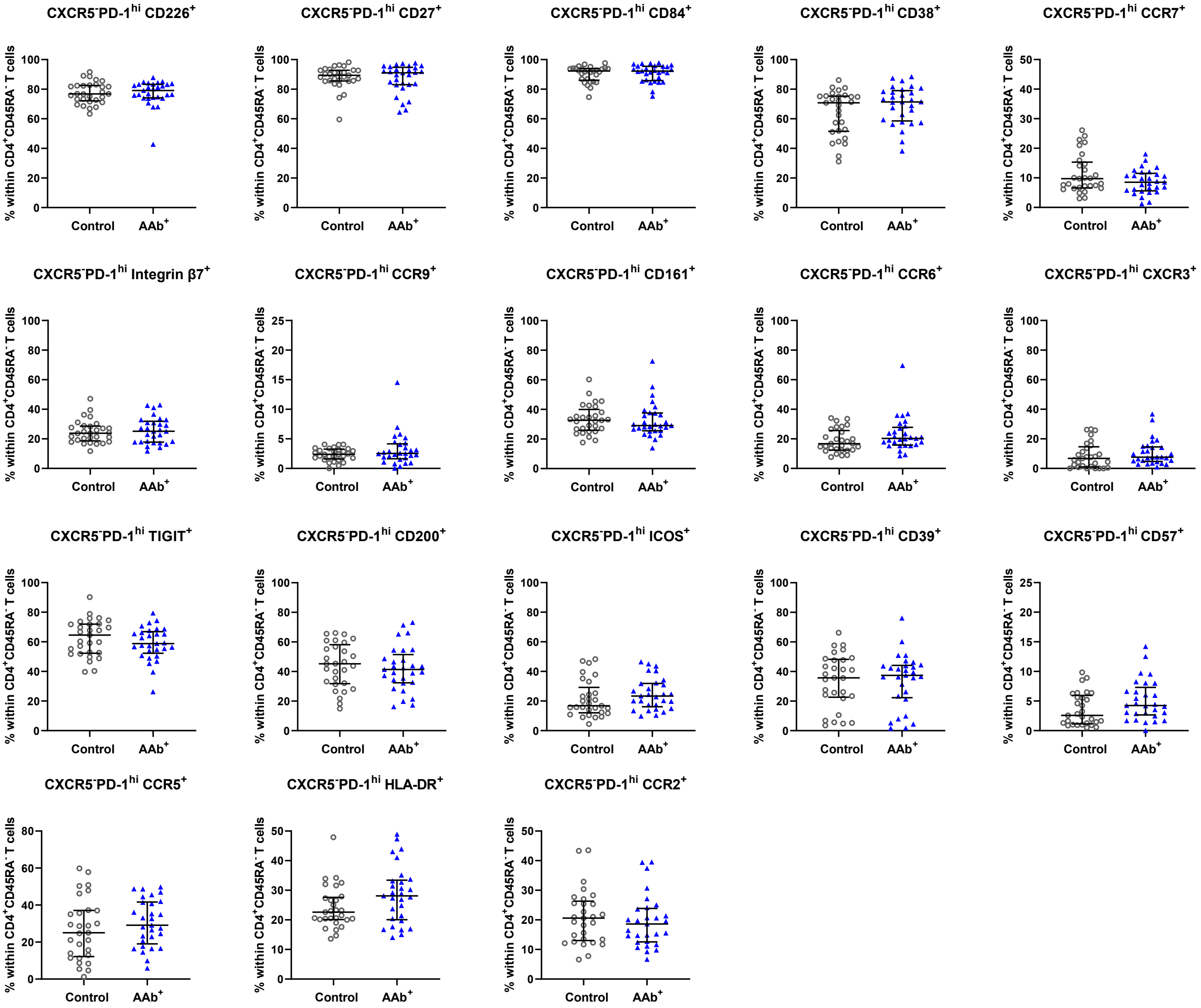

Supporting Figure 5. Expression of 18 surface markers on CXCR5$^-$PD-1^{hi} cTph cells in AAb$^+$ children and controls. Wilcoxon matched-pairs signed rank test was used for statistical testing. After Bonferroni correction, P<0.003 was considered statistically significant. Median with IQR is shown in the figures. Controls are depicted as open grey circles and AAb$^+$ children as solid blue triangles.

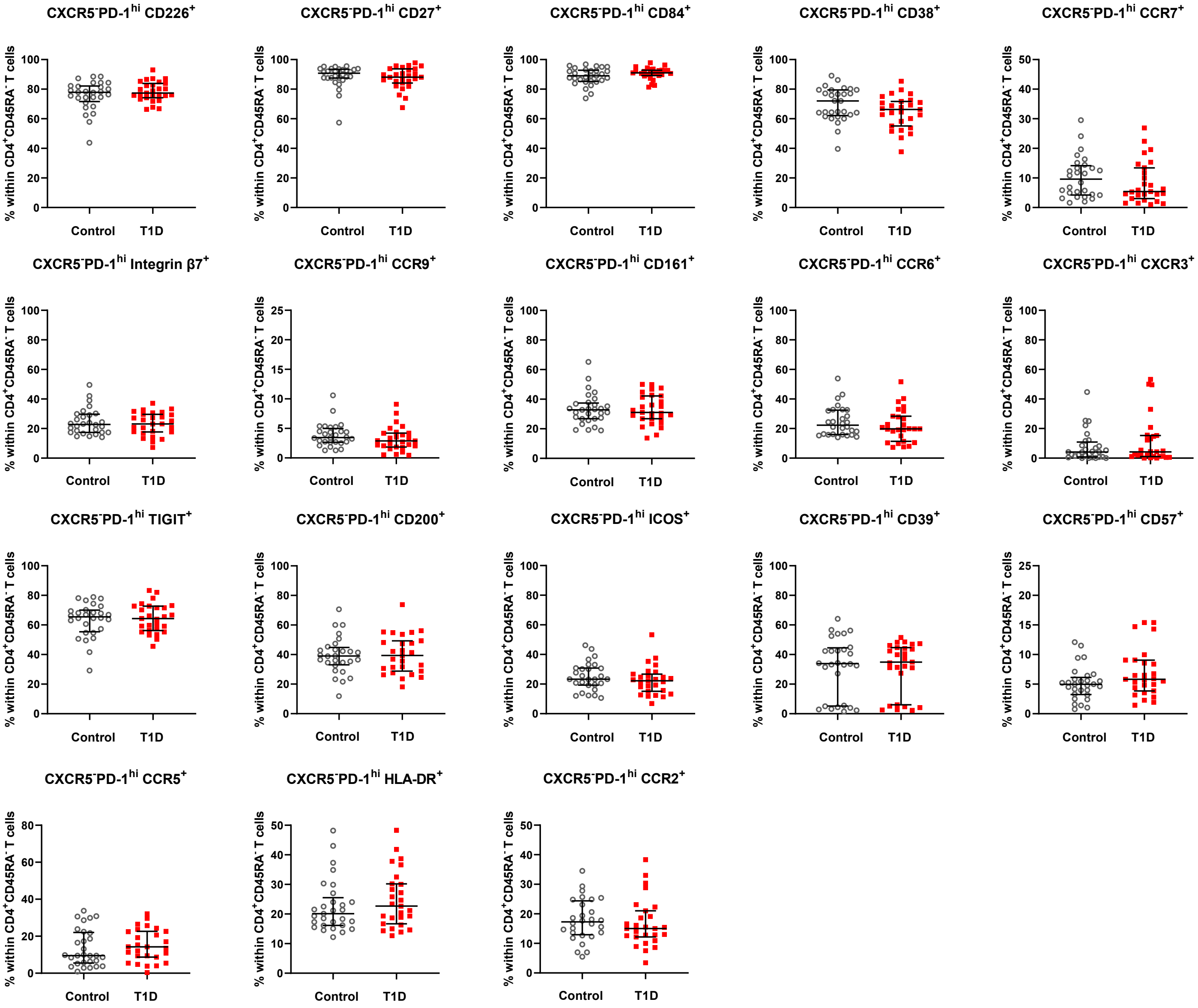

Supporting Figure 6. Expression of 18 surface markers on CXCR5^{-}PD-1^{hi} cTph cells in children with newly diagnosed T1D and controls. Wilcoxon matched-pairs signed rank test was used for statistical testing. After Bonferroni correction, P<0.003 was considered statistically significant. Median with IQR is shown in the figures. Controls are depicted as open grey circles and children with T1D as solid red squares.

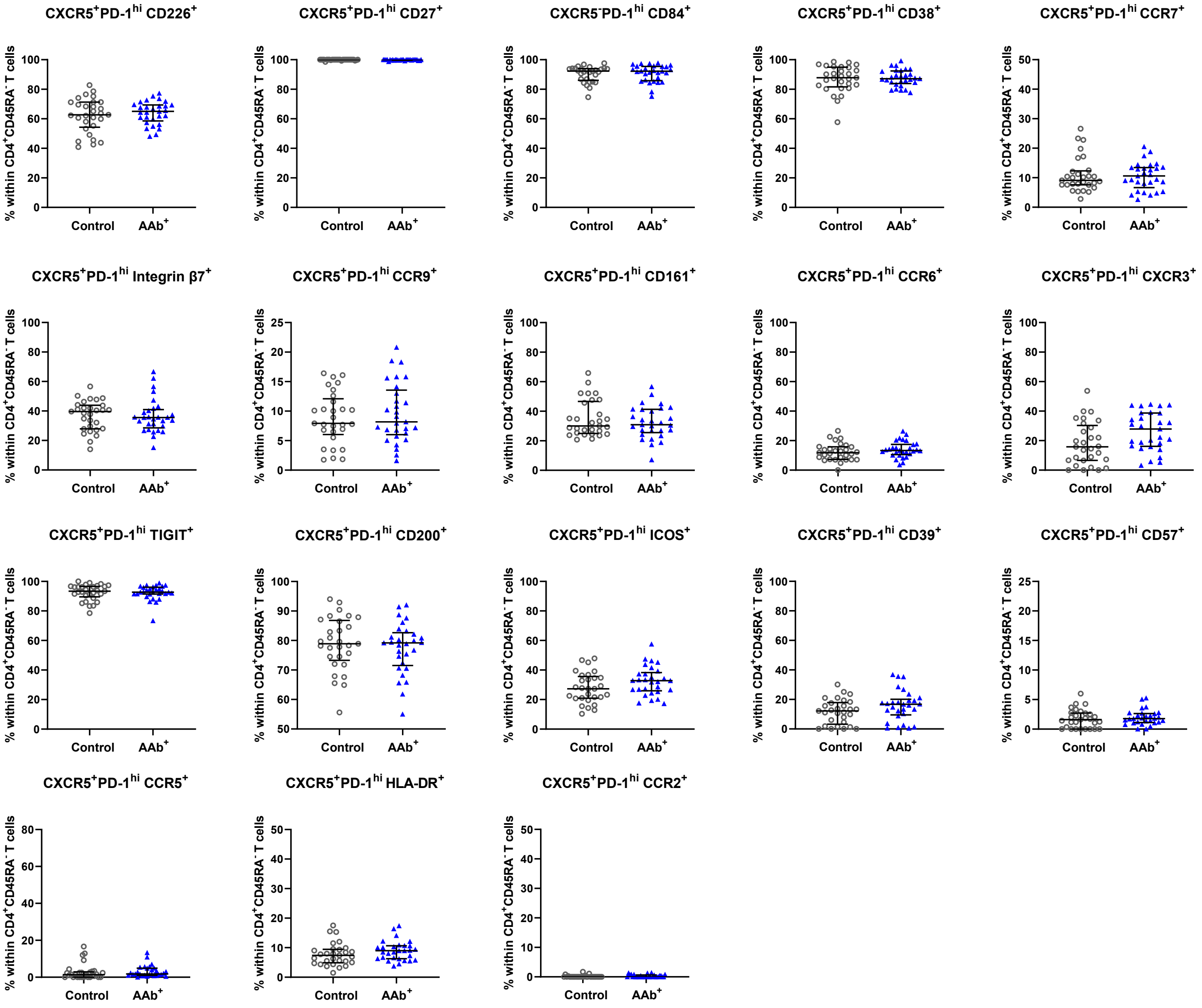

Supporting Figure 7. Expression of 18 surface markers on CXCR5^{+}PD-1^{hi} cTfh cells in AAb^{+} children and controls. Wilcoxon matched-pairs signed rank test was used for statistical testing. After Bonferroni correction, $P<0.003$ was considered statistically significant. Median with IQR is shown in the figures. Controls are depicted as open grey circles and AAb^{+} children as solid blue triangles.

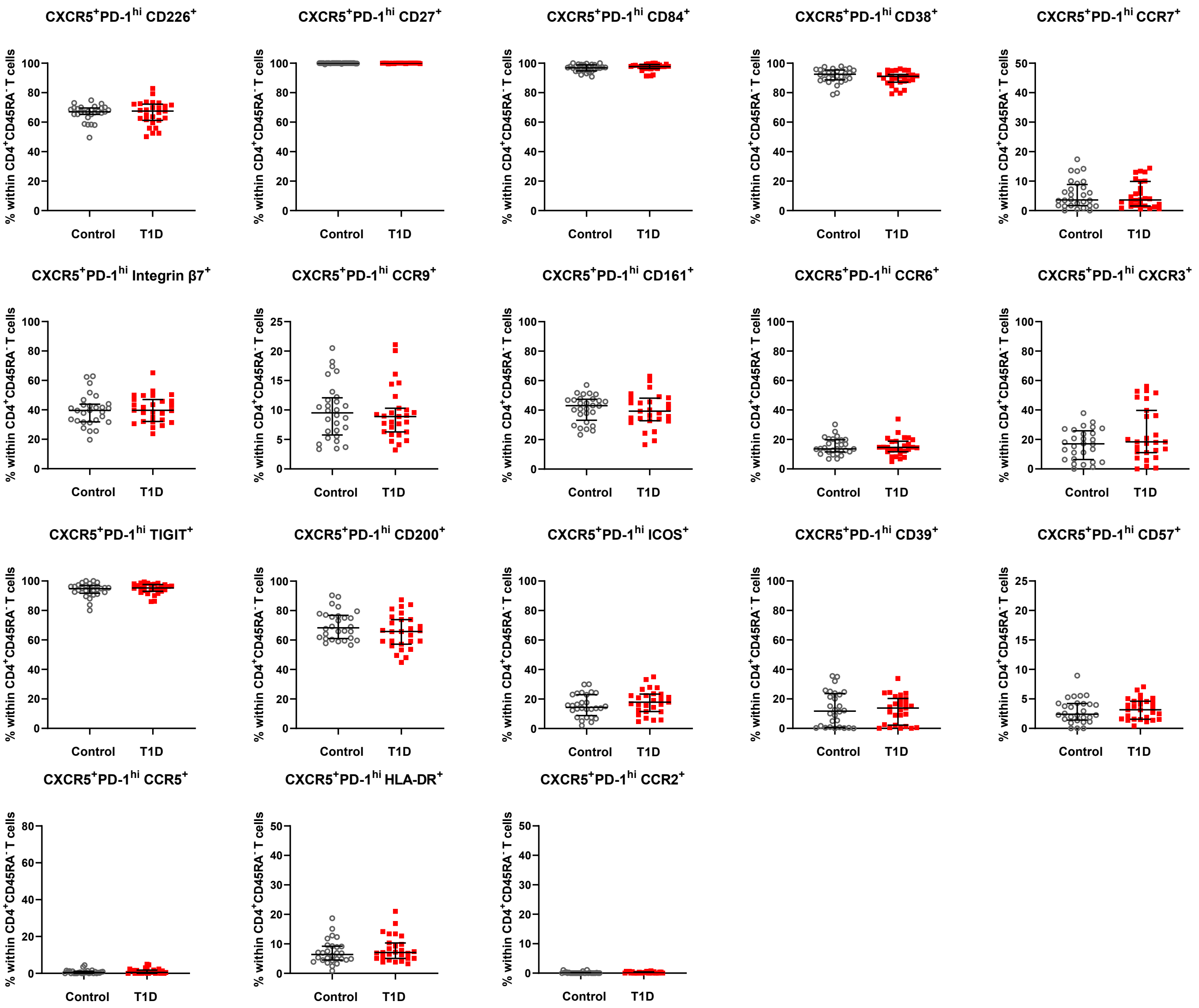

Supporting Figure 8. Expression of 18 surface markers on CXCR5^{+}PD-1^{hi} cTfh cells in children with newly diagnosed T1D and controls. Wilcoxon matched-pairs signed rank test was used for statistical testing. After Bonferroni correction, $p<0.003$ was considered statistically significant. Median with IQR is shown in the figures. Controls are depicted as open grey circles and children with T1D as solid red squares.

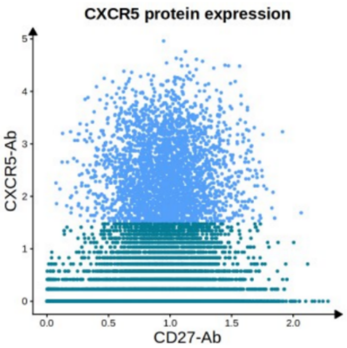

Supporting Figure 9. CXCR5 protein (Ab) expression depicted as scatter plot in the single-cell mutltiomics experiment. CXCR5 protein expressing cells were selected using WhichCells command in Seurat for pseudobulk analyses. Normalized expression values >1.5 were considered as CXCR5 positive.

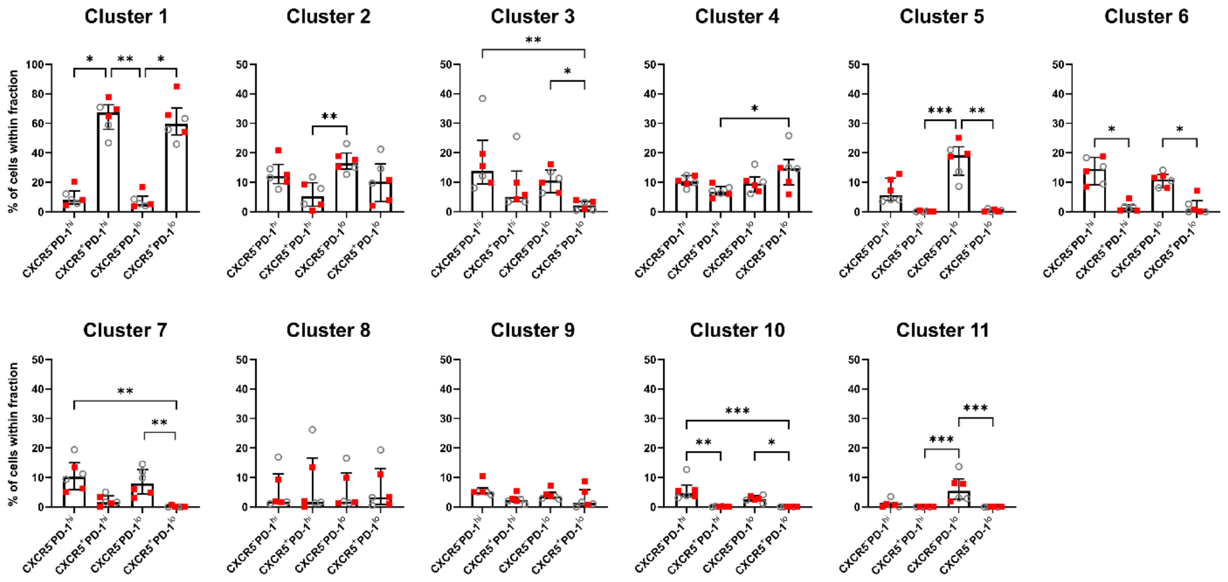

Supporting Figure 10. Frequencies of cells per sample in each cluster localized in the four CXCR5^{-}/$^{+}$PD-1^{hi}/^{lo} cell fractions. Kruskal-Wallis test with Dunn's multiple comparisons was used in the statistical analysis. Controls are depicted as open grey circles and children with newly diagnosed T1D as solid red squares. *P<0.05, **P<0.01, ***P<0.001.

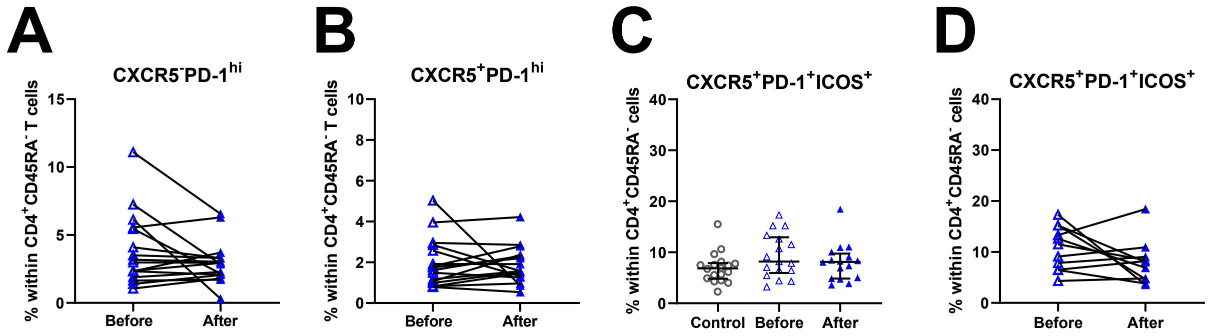

Supporting Figure 11. Comparable frequency of B cell-helper T cells in children before and after seroconversion. Frequency of CXCR5^{-}PD-1^{hi} cTph **(A)** or CXCR5^{+}PD-1^{hi} cTfh **(B)** frequencies in pairwise longitudinal analysis in children before and after seroconversion (n=16), the analysis contains also individuals who did not progress to T1D during the follow-up. Frequency of CXCR5^{+} PD-1^{+}ICOS^{+} activated cTfh cells in healthy controls (n=18) and children before (n=17) and after (n=16) seroconversion **(C)** or before and after seroconversion in children who later progressed to T1D (n =11) **(D)**. Wilcoxon matched-pairs signed rank test was used for statistical analysis when two groups were compared and Kruskal-Wallis with Dunn's multiple comparisons test when three groups were compared. Controls are depicted as open grey circles, children before seroconversion as open blue triangles and children after seroconversion (AAb^{+}) as solid blue triangles.

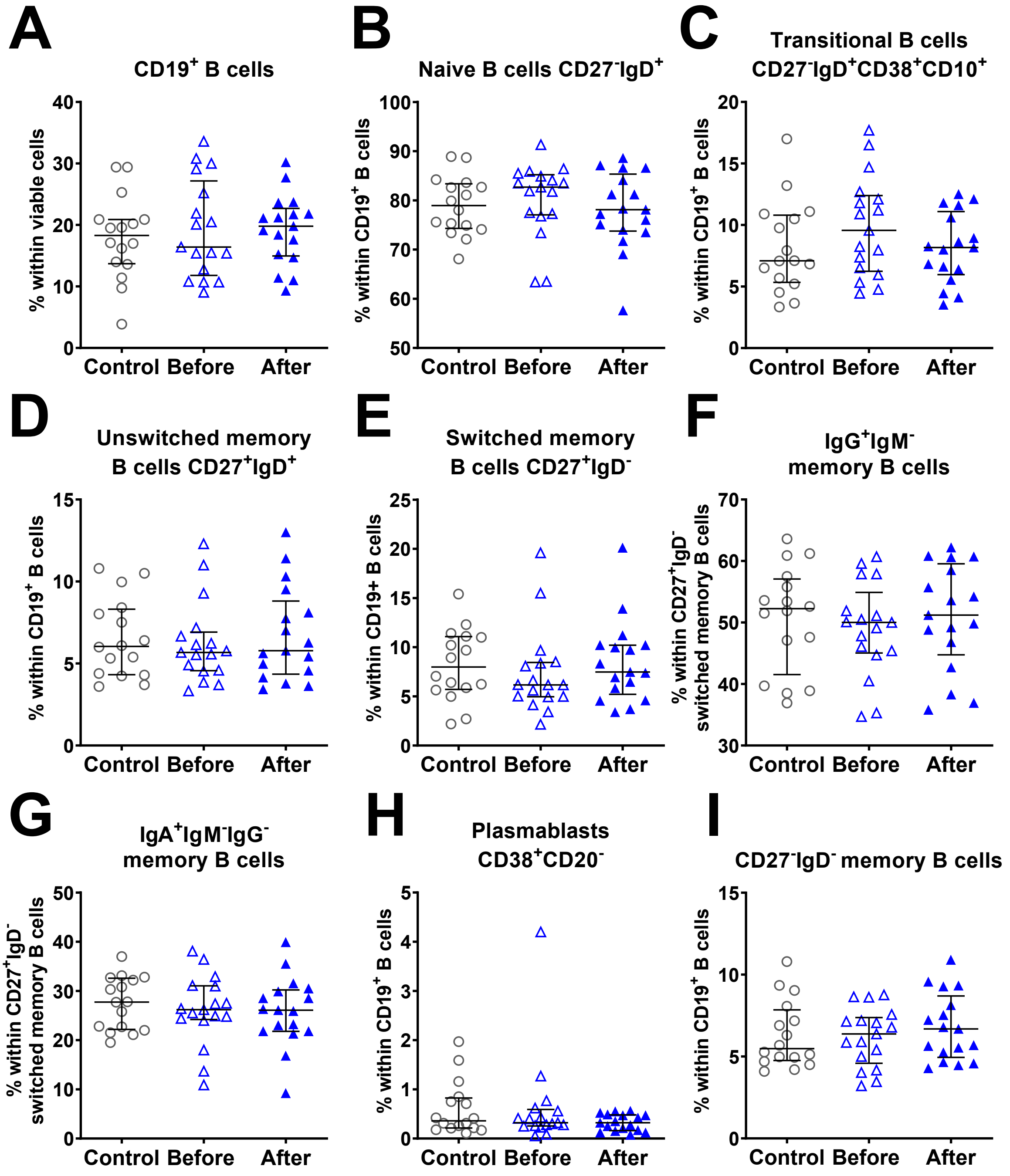

Supporting Figure 12. B cells frequencies are comparable in children before and after seroconversion. Frequencies of $CD19^+$ B cells **(A),** naïve B cells **(B),** transitional B cells **(C),** unswitched memory B cells **(D),** switched memory B cells **(E),** IgG^+ memory B cells **(F),** IgA^+ memory B cells **(G),** plasmablasts **(H)** and $CD27^-IgD^-$ memory B cells **(I)** in healthy controls and children before and after AAb seroconversion. Kruskal-Wallis with Dunn's multiple comparisons test was used in statistical testing. Control n=16, before n=17, after n=17. Controls are depicted as open grey circles, children before seroconversion as open blue triangles and children after seroconversion (AAb^+) as solid blue triangles.

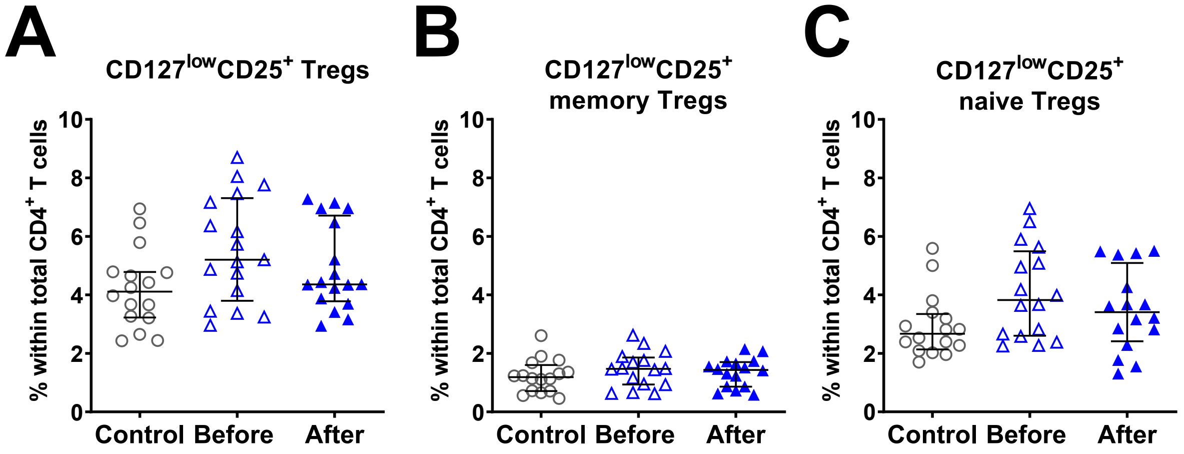

Supporting Figure 13. Comparable frequency of regulatory T cells (Tregs) in children before and after seroconversion. Frequency of total **(A),** memory **(B)** and naïve $CD4^+CD127^{low}CD25^+$ Tregs **(C)** in healthy controls and children before and after AAb seroconversion. Kruskal-Wallis with Dunn's multiple comparisons test was used in statistical testing. In A, control n=16, before n=17, after n=17. In B and C, control n=16, before n=16, after n=16. Controls are depicted as open grey circles, children before seroconversion as open blue triangles and children after seroconversion (AAb^+) as solid blue triangles.

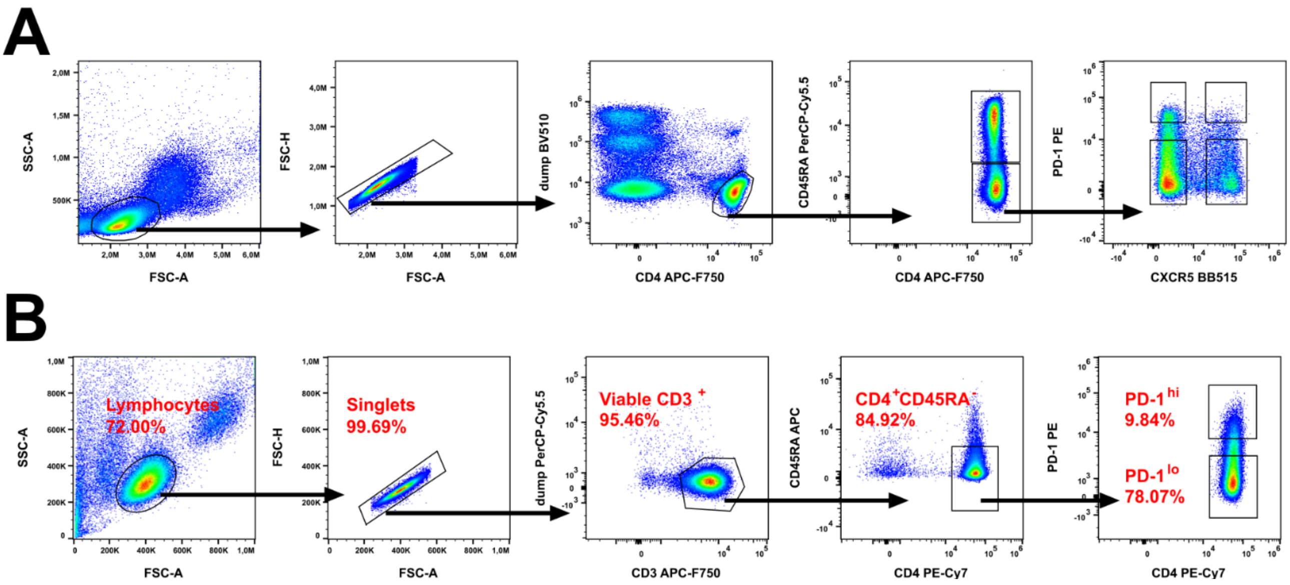

Supporting Figure 14. Gating strategy for flow cytometry. (A) The following gating strategy was used for multiparameter flow cytometry analyses. First, lymphocytes were gated according to their morphology with forward scatter (FSC-A) and side scatter (SSC-A) properties, and doublets were excluded using FSC-A and FSC-H. Viable CD4$^+$ T cells were gated excluding dead cells and CD14, CD56, CD16, and CD19 (all in BV510). Memory CD4$^+$ T cells were identified as CD4$^+$CD45RA$^-$ and subsequently four different fractions were gated according to CXCR5 and PD-1 expression. cTph cells were identified as CXCR5$^-$PD-1^{hi} and their cTfh counterpart as CXCR5$^+$PD-1^{hi}. Phenotypical markers were gated according to fluorescence minus one controls. **(B)** Representative figure of sorting setup for one donor for single-cell multiomics experiment. First, lymphocytes were gated according to their morphology with forward scatter (FSC-A) and side scatter (SSC-A) properties, and doublets were excluded using FSC-A and FSC-H. Viable CD3$^+$ T cells were gated excluding dead cells (7-AAD$^+$), CD14$^+$, CD56$^+$, CD16$^+$ and CD19$^+$ (all in PerCP-Cy5.5). Memory CD4$^+$ cells were identified as CD4$^+$CD45RA$^-$ and PD-1^{hi} and PD-1^{lo} cells were subsequently two-way sorted.

Supporting Table 12. Characteristics of pediatric cohort 1.

	**Controls**	**AAb⁺ children**	**Children with newly diagnosed T1D**
**N**	57	29	27
**Mean age at sampling, SD (y)**	8.2 ± 3.8	8.6 ± 3.6	8.0 ± 3.9
**Age range (y)**	2.0–15.0	2.8–15.3	1.9–14.6
**Time from sampling to diagnosis mean ± SD (y)**	N/A	1.9 ± 1.0	N/A
Female	40% (23/57)	45% (13/29)	33% (9/27)
Male	60% (34/57)	55% (16/29)	67% (18/27)
HLA class II genotype			
DQ2/DQ2 or DQ2/X	23% (13/57)	14% (4/29)	22% (6/27)
DQ8/DQ8 or DQ8/X	53% (30/57)	52% (15/29)	59% (16/27)
DQ2/DQ8	23% (13/57)	34% (10/29)	11% (3/27)
X/X	2% (1/57)	-	7% (2/27)
Autoantibodies:			
IAA	ND	41% (12/29)	67% (18/27)
GADA	ND	79% (23/29)	63% (17/27)
IA-2A	ND	66% (19/29)	78% (21/27)
Autoantibody number:			
≤1	N/A	31% (9/29)	26% (7/27)
≥2	N/A	69% (20/29)	74% (20/27)

N/A, not applicable; -, negative; ND, not detected; X, HLA genotype other than DQ2 or DQ8.

Supporting Table 13. Characteristics of pediatric cohort 2.

	Controls	Before seroconversion	After seroconversion
**N**	18	17	16
**Mean age at sampling, SD (y)**	3.6 ± 1.6	2.9 ± 1.3	3.6 ± 1.3
**Age range (y)**	1.5–6.8	1.3–5.6	1.8 ± 6.0
Female	67% (12/18)	41% (7/17)	44% (7/16)
Male	33% (6/18)	59% (10/17)	56% (9/16)
**Progressed to T1D**	N/A	N/A	69% (11/16)
**Time from seroconversion to diagnosis mean ± SD (y)**	N/A	N/A	5.0 ± 1.6
**Time between before and after samples, range (months)**	N/A	N/A	3–13

N/A, not applicable; -, negative.

Supporting Methods

Library preparation and alignment, quantification and quality control of single-cell multiomics data

Reverse transcription, template switching, TCR CDR3 extension, and Exonuclease I treatment were performed, according to manufacturer's instruction. cDNA was then amplified using PCR1 primers for selected immune genes, AbSeq and Sample Tags and TCR products. PCR1 products were purified using AMPure XP beads (Beckman Coulter). Purified mRNA, Sample Tag and TCR products were amplified further (PCR2) and subsequently purified using AMPure XP beads. Index PCRs were performed for each modality to prepare the final libraries. The concentration and fragment size distribution of each index PCR library was assessed with Qubit (Qubit® dsDNA HS Assay Kit, Life Technologies) and Bioanalyzer (High Sensitivity DNA kit, Agilent). The final libraries were pooled at 2 nM concentration. 20% PhiX spike-in was used, and libraries were sequenced using SP 300 cycles kit cycle kit on Novaseq 6000 Illumina (FFGC Turku, Finland).

The sequence reads were aligned to the reference genome hg38 using the BD Rhapsody™ T Cell Expression Panel Hs FASTA file (https://scomix.bd.com/hc/en-us/articles/360044810091-Reference-files-fasta, accessed on 27.10.22) and Gencode annotation (version 26) *via* SevenBridges Genomics server (https://www.sevenbridges.com/) employing the BD Rhapsody Targeted analysis pipeline (revision13). In total, 14,005 single cells were obtained after sequencing the PD-1^{hi} (6911 cells) and PD-1^{lo} (7094 cells) fractions. Cells without sample tag information, multiplets, and <26 detected genes were filtered out, resulting in 12,916 cells for downstream analysis. Genes expressed in <5 cells were removed from the dataset, leaving 244 genes were for the analysis.

Single-cell multiomics data analysis

Seurat [1] version 4.1.3 was used for the single-cell multiomics data analysis. Raw gene counts were normalized using the default parameters of the NormalizeData function, *i.e.*, for each cell, gene-wise counts were divided by the total library size to account for sequencing depth differences, scaled by 10,000, and finally log1p-transformed for variance stabilization. Canonical correlations analysis (CCA) was performed to integrate the PD-1^{hi} and PD-1^{lo} data fractions by applying the FindIntegrationAnchors and IntegrateData functions. AbSeq protein expression data was normalized using centered log ratio (CLR) implemented in the NormalizeData function (normalization.method = "CLR"). A joint low-dimensional space representation of mRNA and protein data was created using the Weighted Nearest Neighbor (WNN) method using FindMultiModalNeighbors. Cell clusters were identified using the WNN graph by running the default Louvain algorithm *via* the FindClusters function (resolution = 0.5). Uniform Manifold Approximation and Projection (UMAP) dimensional reduction was generated with RunUMAP function.

Pseudobulk analysis

Counts per sample were aggregated using decoupleR [2] (version 1.6.0) and normalized for differences in sequencing depth to counts per million (CPM) in EdgeR [3–5] version 3.40.0 . Filtering of low expressed genes was performed using the FilterByExpr() function, resulting in 196 genes remaining for the subsequent analysis. After filtering, library sizes were recomputed, and counts normalized using the trimmed mean of M-value (TMM) normalization. PCA plot was generated using the plotMDS (gene.selection = "common") function in edgeR. For differential gene and surface protein expression testing, the generalized linear model quasi-likelihood (QL) pipeline was used [6]. The glmTreat test was applied to test for differential expression relative to FC > 1.5. In addition, pseudobulk analysis was conducted also in parallel using DESeq2 implemented in Seurat 5 [7].

Shortly, AggregateExpression function was used to create pseudobulk samples and then FindMarkers function was used to run the differential expression analysis using DESeq2.

Gene ontology (GO) enrichment analysis

Gene ontology (GO) enrichment analysis was conducted using enrichR [8] (version 3.4) in R [9] using term "GO_Biological_Process_2023" for $CXCR5^{-}PD\text{-}1^{hi}$, $CXCR5^{+}PD\text{-}1^{hi}$, $CXCR5^{-}PD\text{-}1^{lo}$ and $CXCR5^{+}PD\text{-}1^{lo}$ cell fractions. Shortly, significantly upregulated genes in each fraction were selected and function enrichr was used to calculate the enriched terms per fraction using these upregulated gene lists.

Supporting Methods References

[1] Y. Hao, S. Hao, E. Andersen-Nissen, W.M. Mauck, S. Zheng, A. Butler, M.J. Lee, A.J. Wilk, C. Darby, M. Zager, P. Hoffman, M. Stoeckius, E. Papalexi, E.P. Mimitou, J. Jain, A. Srivastava, T. Stuart, L.M. Fleming, B. Yeung, A.J. Rogers, J.M. McElrath, C.A. Blish, R. Gottardo, P. Smibert, R. Satija, Integrated analysis of multimodal single-cell data, Cell 184 (2021) 3573-3587.e29. https://doi.org/10.1016/j.cell.2021.04.048.

[2] P. Badia-I-Mompel, J. Vélez Santiago, J. Braunger, C. Geiss, D. Dimitrov, S. Müller-Dott, P. Taus, A. Dugourd, C.H. Holland, R.O. Ramirez Flores, J. Saez-Rodriguez, decoupleR: ensemble of computational methods to infer biological activities from omics data, Bioinforma. Adv. 2 (2022) 1–3. https://doi.org/10.1093/bioadv/vbac016.

[3] M.D. Robinson, D.J. McCarthy, G.K. Smyth, edgeR: A Bioconductor package for differential expression analysis of digital gene expression data, Bioinformatics 26 (2009) 139–140. https://doi.org/10.1093/bioinformatics/btp616.

[4] D.J. McCarthy, Y. Chen, G.K. Smyth, Differential expression analysis of multifactor RNA-Seq experiments with respect to biological variation, Nucleic Acids Res. 40 (2012) 4288–

4297. https://doi.org/10.1093/nar/gks042.

[5] Y. Chen, L. Chen, A.T.L. Lun, P.L. Baldoni, G.K. Smyth, edgeR 4.0: powerful differential analysis of sequencing data with expanded functionality and improved support for small counts and larger datasets, BioRxiv (2024) 2024.01.21.576131. https://www.biorxiv.org/content/10.1101/2024.01.21.576131v1%0Ahttps://www.biorxiv.org/content/10.1101/2024.01.21.576131v1.abstract.

[6] Y. Chen, A.T.L. Lun, G.K. Smyth, From reads to genes to pathways: Differential expression analysis of RNA-Seq experiments using Rsubread and the edgeR quasi-likelihood pipeline, F1000Research 5 (2016) 1–51. https://doi.org/10.12688/F1000RESEARCH.8987.2.

[7] Y. Hao, T. Stuart, M.H. Kowalski, S. Choudhary, P. Hoffman, A. Hartman, A. Srivastava, G. Molla, S. Madad, C. Fernandez-Granda, R. Satija, Dictionary learning for integrative, multimodal and scalable single-cell analysis, Nat. Biotechnol. 42 (2024) 293–304. https://doi.org/10.1038/s41587-023-01767-y.

[8] M. V. Kuleshov, M.R. Jones, A.D. Rouillard, N.F. Fernandez, Q. Duan, Z. Wang, S. Koplev, S.L. Jenkins, K.M. Jagodnik, A. Lachmann, M.G. McDermott, C.D. Monteiro, G.W. Gundersen, A. Maayan, Enrichr: a comprehensive gene set enrichment analysis web server 2016 update, Nucleic Acids Res. 44 (2016) W90–W97. https://doi.org/10.1093/nar/gkw377.

[9] R Core Team, A language and environment for statistical computing., (2021). https://www.r-project.org/.
